# A systematic review of Inconel 939 alloy parts development via additive manufacturing process

**DOI:** 10.1016/j.heliyon.2024.e25506

**Published:** 2024-02-01

**Authors:** Syed Abbas Raza, Olcay Ersel Canyurt, Hüseyin Kürşad Sezer

**Affiliations:** a Department of Mechanical Engineering, Faculty of Engineering, Gazi University, Eti Mah. Yukselis Sk. No: 5, 06570, Maltepe, Ankara, Turkey; b Additive Manufacturing Technologies Research and Application Center-EKTAM, Gazi University, Saray OSB Mahallesi, Uzay ve Havacılık OSB Küme Evleri, No:62 Kahramankazan, Ankara, Turkey; c Department of Industrial Design Engineering, Faculty of Technology, Gazi University, Emniyet Mah. Bandırma Cad. No: 6, 06560, Yenimahalle, Ankara, Turkey

**Keywords:** IN939, Additive manufacturing, Heat treatment, Tensile testing, Creep, Fatigue, Microstructure, Phase analysis

## Abstract

IN939 is a modern class of nickel-based superalloys designed for continuous operational sustenance at elevated temperatures owing to their excellent combination of fatigue, creep, and corrosion resistance. This unique performance of IN939 is associated with the composition of this alloy, along with specific post-processing treatments such as solution treatment and aging, giving rise to features such as the presence of γ’ residues, as well as the presence of MC and M_23_C_6_ carbides. This also includes the absence of the eutectic and incipient melting phases. For this alloy, the primary part development is by the powder bed fusion process using a laser powder bed fusion machine. At the same time, a solo study highlights the use of an EB-PBF machine for the synthesis. The AM development process of these alloys is hindered by machine parameters, which have been found ineffective in isolation to obtain a fully dense structure with desired properties. The purpose of these parameters is to improve their core properties while minimizing defects associated with powder metallurgy routes, such as porosity, detrimental precipitates, grain anisotropy, etc. This study aims to provide an overview of the advancements in research related to IN939, explicitly focusing on the benchmarks achieved through additive manufacturing techniques. We have discussed the work performed in this area, compared the results of different studies, and identified the gaps in current research. By doing so, we aim to provide a comprehensive understanding of the potential of IN939 and its applications in extreme environments.

## 1 Introduction

### 1.1 Overview of Inconel 939

**Fig. 1:**
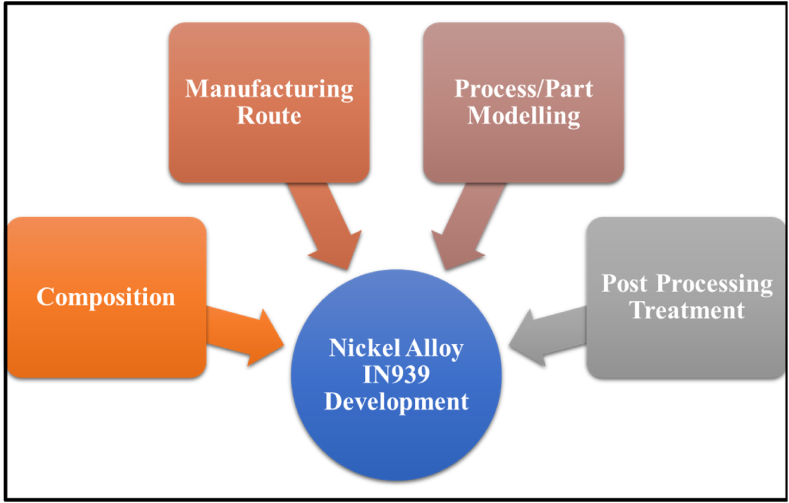
The key aspects of developing IN939 for parts.

**Fig. 2:**
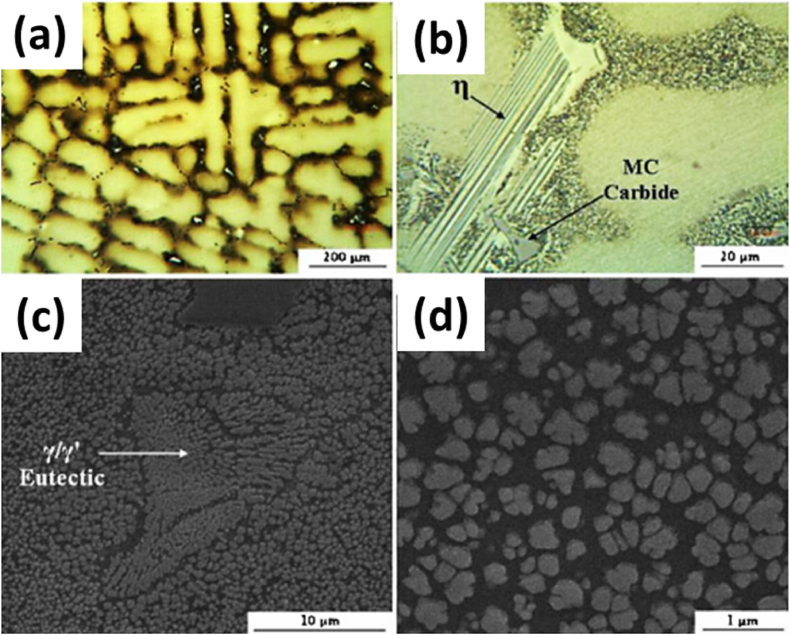
The microstructure of the cast specimen showing (a) The dendritic microstructure, (b). the MC phase and η phase, (c) the γ/γ′ phase, and (d) rose like γ′ precipitates [3].

IN939 is a class of advanced, high-temperature nickel-based alloys that modify the Waspaloy system [1]. It is a high-temperature alloy with a service temperature of up to 1123 K (850 °C). It is preferred for high-temperature service applications due to its superior creep strength, corrosion, and oxidation resistance owing to its composition **[**Table 1**]**. It has primarily been utilized in the form of cast alloy for the manufacturing of gas turbine blades/vanes as well as castings for turbines and burner nozzles, as seen from the prerequisites highlighted in Fig. 1. The strengthening mechanism of the alloy is due to the combination of the dendritic gamma solid solution matrix (γ) with a dispersion of gamma-prime precipitates (γ′) Fig. 2 (d), which enhance its mechanical properties [2,3]. The IN939 is typically produced as a solid cast and contains a large volume fraction of γ′ phase Fig. 2 (a), which can reach up to 30–40 % after heat treatment, and a γ-γ′ eutectic phase, which results from slower cooling, as can be seen in Fig. 2 (c). This is ratified by solution treatment of the part to create a super-saturated solid solution, which is then aged to produce γ’ strengthening phase [4].

**Table 1:** The general composition of IN939 alloy [20].

Element	Composition (wt.%)	Element	Composition (wt.%)	Element	Composition (wt.%)
**Al**	Max. 2.0	**Fe**	Max 0.5	**S**	Max. 0.002
**Ni**	Bal.	**Mn**	Max. 0.5	**B**	Max. 0.015
**C**	Max. 0.15	**W**	Max. 3.0	**Si**	Max. 0.5
**Co**	18.0–20.0	**Nb**	Max. 1.2	**Ti**	Max. 4.0
**Cr**	20.0–23.0	**Ta**	Max. 2.0	**Zr**	Max. 0.15

**Fig. 3:**
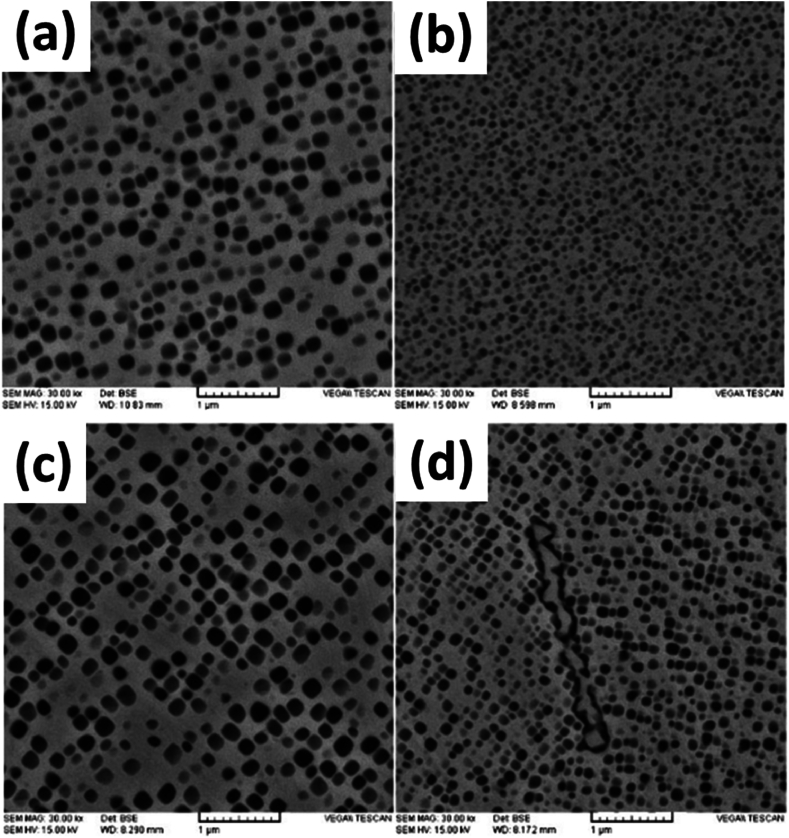
The microstructure of the cast specimen showing microstructures of wrought IN939 alloy after (a) Type A heat treatment, (b). Type B heat treatment, (c) Type A after 1500h aging at 800 °C, and (d) Type B after same aging [3].

The cast alloy route for making IN939 is simple. The alloy is annealed, followed by a single-step aging process to form gamma prime (γ′) precipitates in the matrix. This can be seen in the microstructure demonstrated in Fig. 3 (a-d) where the different sets provide precipitation as a result of different aging treatments in the work of Jahangiri [3]. Solution treatment is carried out in the as-cast alloys in multiple steps at a temperature of 1160 °C. The purpose of this treatment is to dissolve the γ’ sediments, which occur above 1100 °C and are formed in the matrix during the solidification process. On the other hand, aging treatment of the alloy is carried out in the range of 850 °C to 900⁰ [5].

**Fig. 4:**
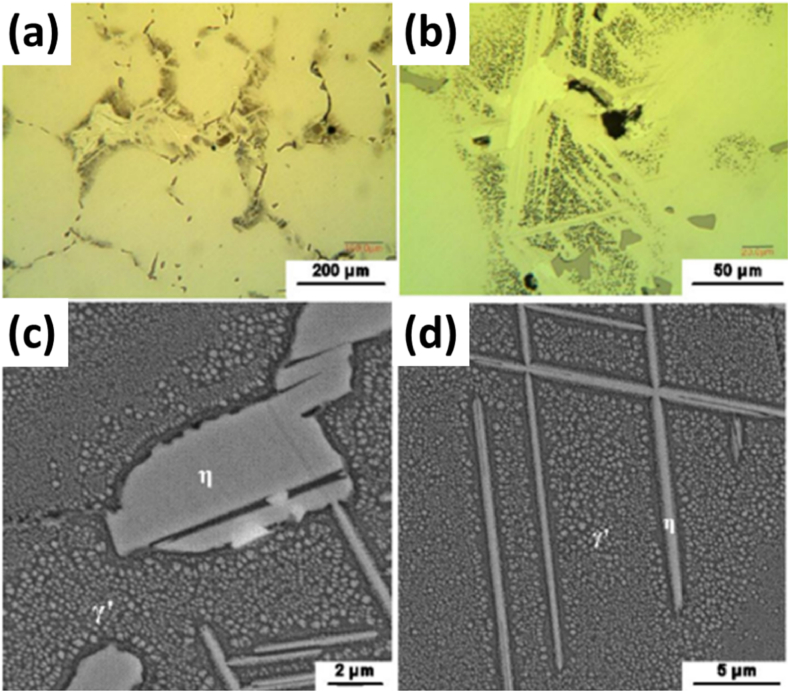
The microstructure of annealed specimens at 4h at 1100⁰ followed by water quenching with (a, b) optical microstructures with different magnifications; (c, d) SEM microstructure from different locations of specimens [33].

**Fig. 5:**
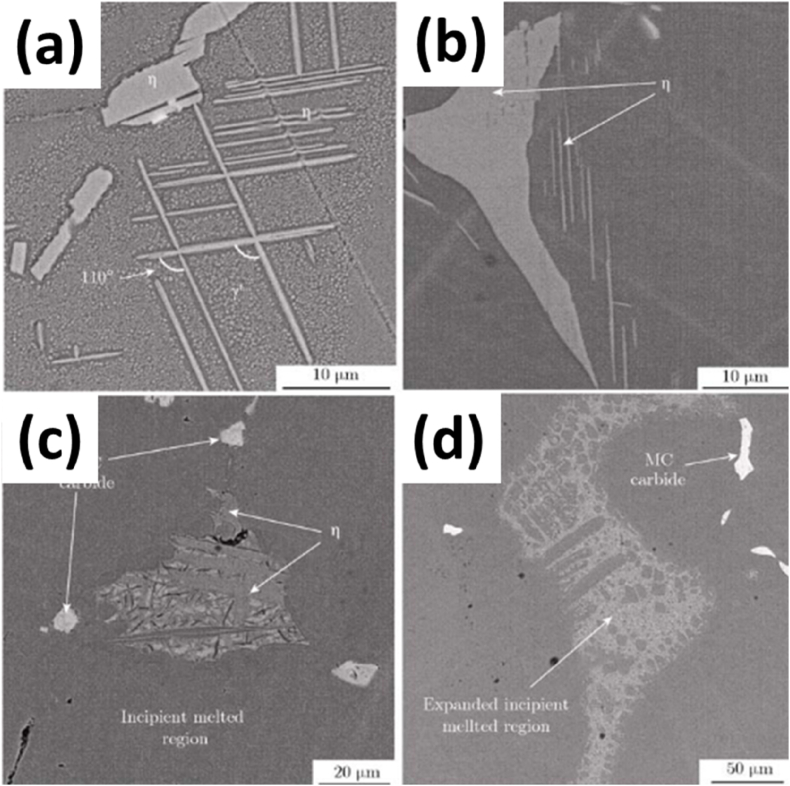
SEM microstructures of specimens annealed at different temperatures for 4 h followed by water quenching: (a) 1100 °C; (b) 1125 °C; (c) 1150 °C; (d) 1250 °C [8].

In addition to removing the gamma prime phase (γ′), solution treatment is necessary to eliminate the detrimental eat (η) phase from the matrix. The η phase impedes the elevated temperature creep properties of the alloy in focus and typically dissolves at a much higher temperature (above 1150 °C) (Fig. 4(a and b)). The η phase exists in the form of platelets, which are formed at the periphery of the γ/γ’ eutectic during the casting process, as seen in Fig. 4 c, d). The η phase is within a specific {111} plane of the matrix. Generally, it has a Ni_3_Ti-type distribution, with no solubility of other elements. However, this statement contradicts research findings [4,6]. Apart from growing only in a specific plane of the matrix, the η phase plates cluster in small groups, with an angle of 110⁰ measured in Fig. 5 (a). Furthermore, solution annealing at increasingly high temperatures leads to the formation of an incipient melting phase along with the presence of η-phase close to the incipient region Fig. 5 (b-d).

**Fig. 6:**
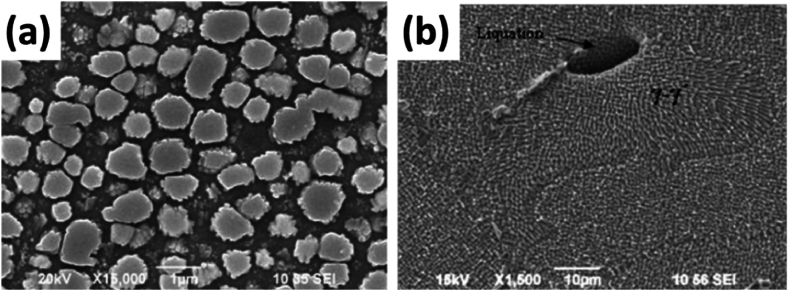
SEM microstructures of (a) Overaged microstructure of γ′, (b) micrograph of eutectic γ-γ′ incipient melting [20].

On the other hand, the γ′ phase exists initially in a cubical form and becomes spherical due to coarsening during the aging treatment. In addition to the volume fraction of precipitates, their morphology must be considered, as seen from the overaged sample micrograph in Fig. 6 (a). In the case of the γ precipitates, their presence is influenced by the heat treatment route and the composition of IN939 (manufacturer) selected. Regarding design, it must be highlighted that the presence of Ti and Al elements promotes γ’ precipitation, and thus, their wt.% in the alloy is a crucial factor. Secondly, the aging parameters applied to the alloy after solution treatment are essential to remove both the η phase formed during the solidification of the cast melt and to overcome the incipient melt phase due to the segregation of elements in the cast structure, as seen in Fig. 6 (b) [5,7,8].

**Fig. 7:**
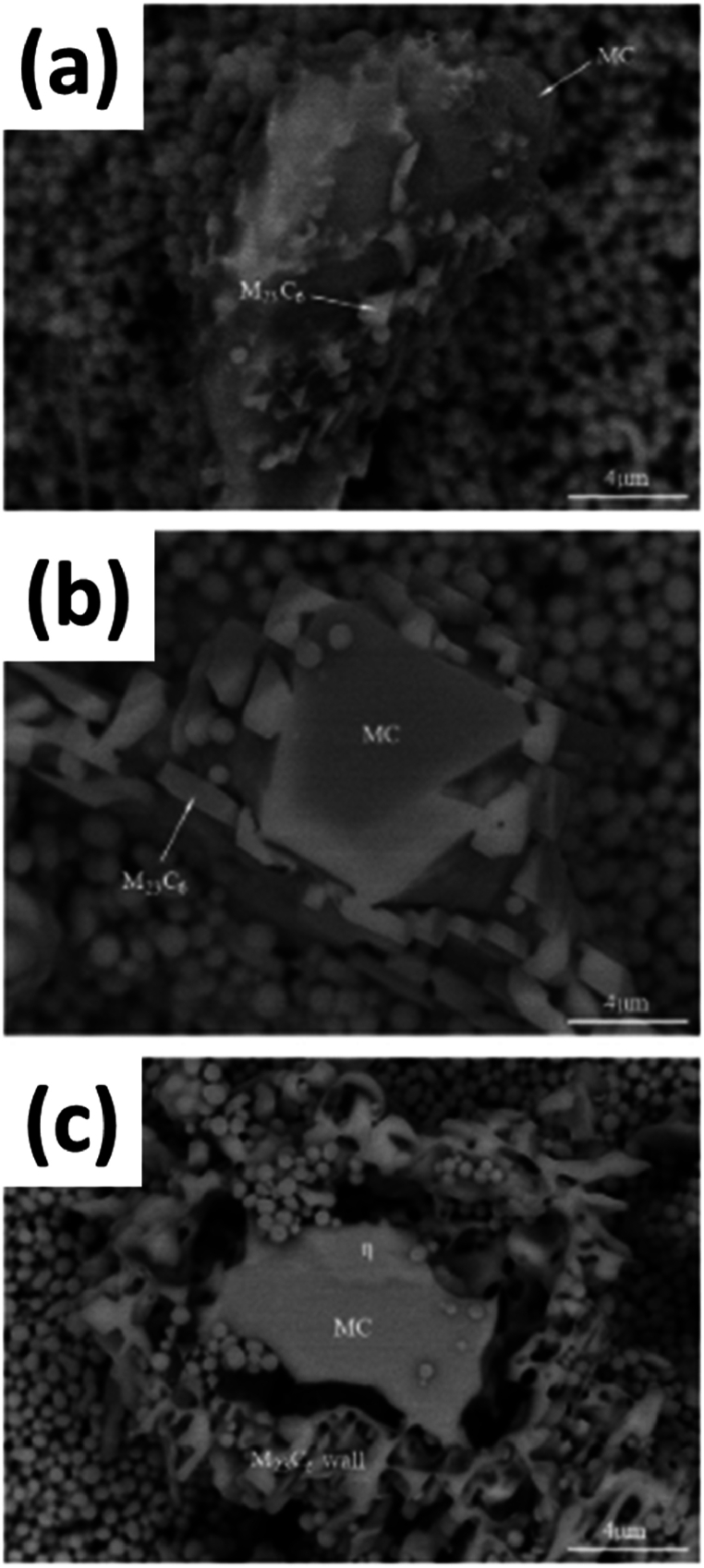
Three-dimensional SEM microstructures of (primary MC degeneration under different thermal exposure conditions: (a) 800 °C/1000 h; (b) 850 °C/5000 h; (c) 850 °C/10,000 h [1].

The strengthening of the IN939 cast alloy is further enhanced by the production of carbides formed during the aging treatment process. There are precisely two types of carbides developed, namely the coarse MC-type carbide and the finer M_23_C_6_-type carbide, which are primarily formed due to the decomposition of the former to produce the latter and γ’, as seen from the observations in Fig. 7 (a-c). These carbides provide strengthening by impeding dislocation motion in the matrix during deformation as well as grain boundary sliding during elevated temperature service, typically at temperatures above 0.5T_m_. When looking at the composition of the IN939 alloy, it can be deduced that it contains a high fraction of carbide-forming elements such as Ti, Ta, and Nb while lacking the element Mo. The carbides, in the absence of Mo, can enhance the fatigue life of the alloy. The symbol M is primarily used to denote a metallic element, which in the case of MC-type carbide is Ti and Cr in the case of M_23_C_7_ carbide [1]. The development of the IN939 alloy for practical applications has been instrumental in resolving problems associated with elevated temperatures. Its mechanical properties are significantly improved by implementing standard heat treatment. An example of this can be seen in the comparison of hardness, which increases from 397 HV for the as-cast alloy to 422 HV for the heat-treated sample. Tensile testing of models under the same heat treatment applied to vanes of land-based turbines showed that, for the as-cast condition, increasing service temperature beyond 700 °C reduced strength but increased ductility. In contrast, in the case of heat-treated samples, an increase in the testing temperature led to a decrease in both the flexibility and strength of the alloy, which was less significant than that of the cast alloy. The casting temperature of the experiment was set at 1400 °C, and the pre-heated temperature of the mold was 800 °C (slightly below modern recommendations). The heat treatment was conducted in two steps: first, a solution treatment was shown in the open air at 1150 °C for 4 h (followed by fast air cooling), followed by aging at 850 °C for 6 h (with air cooling). This aging temperature is below modern recommendations, so the dendritic structure persists. The decrease in strength can be attributed to two phenomena above 750 °C, which include thermally activated cross-slipping and the change in the interaction of dislocation-precipitates from shearing to bypassing precipitates by the dislocations [7]. Indeed, the traditional casting approach to forming alloy IN939 or its components is a time-dependent process during solidification. This process gives rise to chemical and microstructural heterogeneities that result from the directional non-homogeneous cooling solidification of the cast melt structure. Additionally, using traditional cast alloy inevitably leads to material loss and the need for machining and finishing, as it is a top-down process.

### 1.2 Additive manufacturing- overview

Additive manufacturing represents a class of manufacturing using a digital approach to product designing and production, making it, in retrospect, a bottom-up process of manufacturing. The selected process utilized computer-aided drawing (CAD) and automated batch systems to produce parts with complex geometry and tight geometrical tolerance [2,5]. The accuracy of this method lies in the computer-aided manufacturing (CAM) principle whereby the selected part is produced by controlled deposition of the material from which it is to be made. This brings it to the point that additive manufacturing is used for developing parts/components based on all three primary classes of materials: Metals, Polymers, Ceramics, and Composites.

**Fig. 8:**
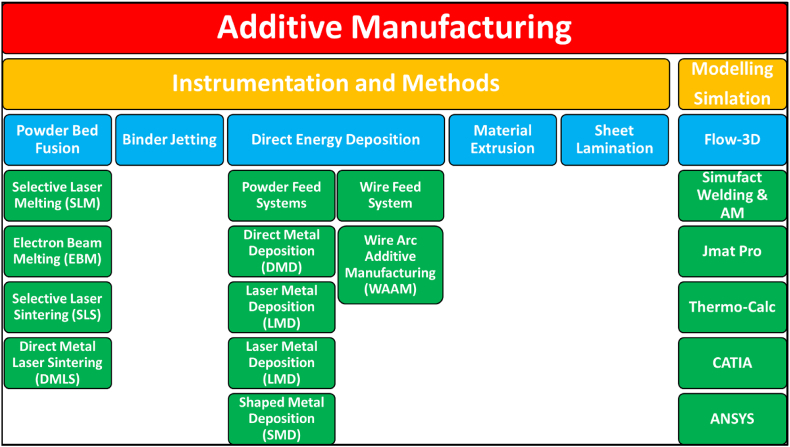
The key categories of Additive Manufacturing and the inclusive software utilization [6].

**Fig. 9:**
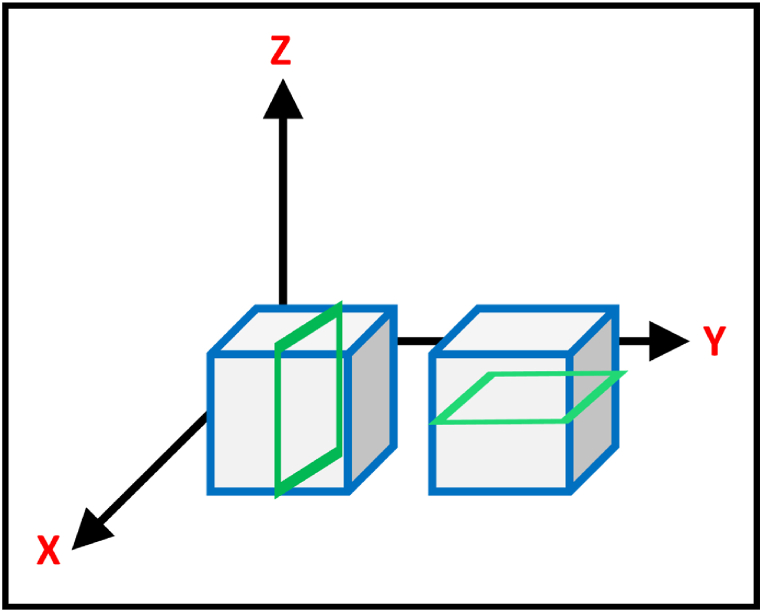
The axis to determine orientation analysis of made samples.

Metal Additive Manufacturing (MAM) utilizes an array of parameter sets to obtain a desirable or at least acceptable part product for use. Several processes have been established for the metal additive manufacturing processes to be utilized in the industry, as shown in Fig. 8. With the use of different equipment, the development of part is performed utilizing building routes as shown in Fig. 9.

Utilizing these raw materials in different conditions allows us to categorize these additive manufacturing processes more simply and highlight their critical operating parameters, benefits, and limits, summarized in Table 2 as per a similar overview provided in earlier works [9,10]. Additive manufacturing processes for hot-temperature materials, particularly metallic, can be classified into the following types.

1. Powder Bed fusion process,
a. Laser powder bed fusion (LPBF)
b. Electron beam-powder bed fusion (EB-PBF)
2. Direct Energy Deposition,
3. Binder Jetting,

**Table 2:** The general comparison of the advantages and disadvantages of selected metal additive manufacturing processes [4,5].

Comparison of key metal additive manufacturing processes			
Instrument Type	Advantages	Disadvantages	Manufacturers
**Electron beam-powder bed fusion**	Reduced Residual stress due to increased temperature	Rough surface Finish	Sciaky Inc.
	Eliminating the need for post-processing temperature	Limited applications of materials	JEOL
	Increased design capabilities	Limited build rate	Retech
	Capacity to produce brittle alloys	A lower variety of materials is possible	General Electric
			Renishaw
			Phoenix System
			FreeMelt
**Laser powder bed fusion**	Great Mechanical Strength	Prone to the development of residual stresses	Aconite 3D
	Superior Part Finish	Additional post-processing treatment is required	3D Systems
	No support required	Slower processing time	Sciaky Inc.
	A large variety of materials is possible.	Required inert atmosphere for metals and alloys	EOS
		Malleability is poor due to defects.	Trumpf
			Xact Metal
			Concept Laser
			FreeMelt
**Direct Energy Deposition**	Extremely high build rate due to the high energy density of the process	High capital cost of equipment and operation	Meltio
	High density and mechanical strength of parts	Lower build resolution leading to poor surface finish	AddUpp
	Wide Materials Range possible	Need for support structures	InssTek
	More significant parts are possible with this process	The high degree of anisotropy and residual stresses	OptoMec
	The more efficient process leads to less waste of raw materials	The large degree of defects in the parts	Lunovu
**Binder Jetting**	No distortion of parts due to the room temperature process	Low control over accuracy and tolerance of dimension	Digital Metal
	Large degree of build volume of parts due to low energy capacity	Need to perform additional sintering and post-processing	ExOne
	The perfect surface finish of parts	Weaker mechanical properties due to internal defects	
	There is no need to provide a support structure for parts	Not suitable for structural components	

As mentioned earlier, all these processes are selective in their applications owing to the differences in the setup, installation, and variation in heating/melting sources and mechanism to form the component from the raw materials [11].

**Fig. 10:**
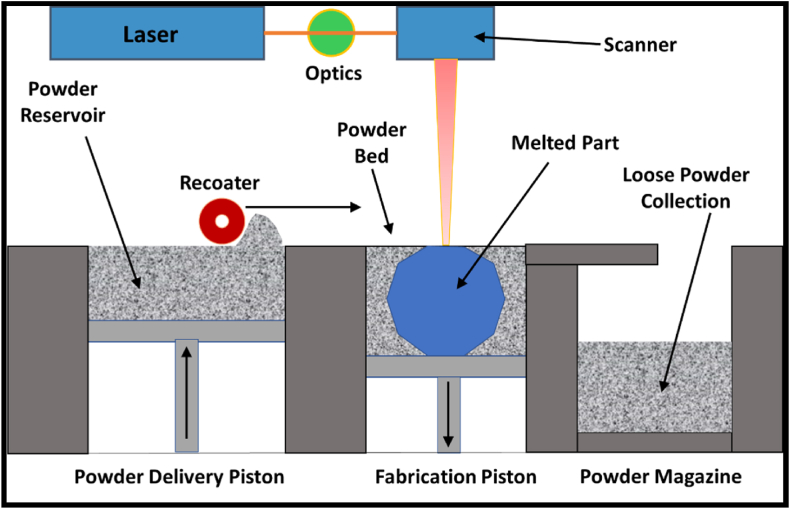
The simple schematic highlighting the selective laser melting process for metal/alloy parts.

**The powder bed diffusion** process is a widely utilized form of additive manufacturing which, as the name suggests, uses a precision melting/fusion source for the layer-by-layer addition of melt material to make the desired part where the feeding to the bed occurs through a separate powder storage tank or hopper [12]. The raw material for melting is placed in a secure chamber bed where layer-by-layer addition takes place as the part fabrication occurs, as shown by the schematic in Fig. 10. These are further categorized as.

• Electron beam powder bed fusion (EPBF)
• Laser Powder Bed Fusion Process (LPBF)

The laser melting process utilizes a series of special lenses to reflect a focused laser beam on the powder bed in the lower half of the setup, whose motion adjusts by the orientation of the lens. The piston system underneath the bed allows for the lateral and longitudinal movement of the bed to shape the part geometry, as highlighted in the CAD file. The chamber is completely enclosed or sealed during operation and purged with inert gas to avoid oxidation/contamination of the metallic/alloy powders during the melting process.

**Fig. 11:**
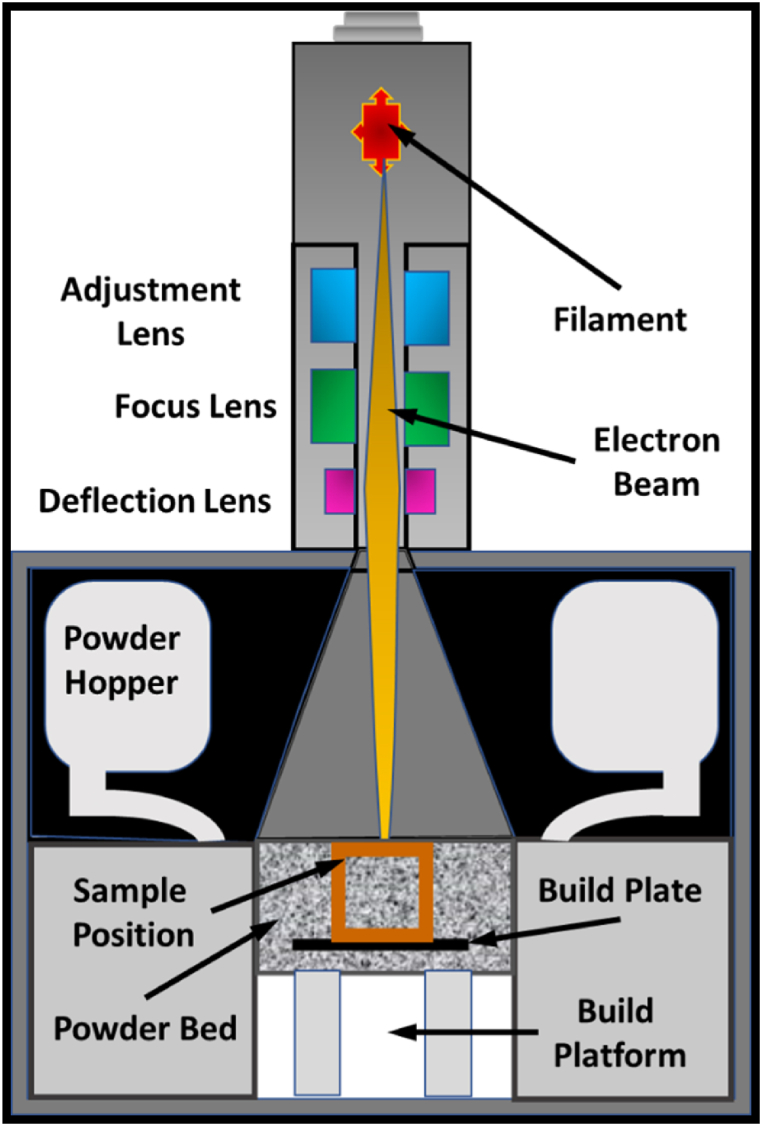
The schematic process of electron beam melting process.

On the other hand, EB-PBF is a process that is more commonly associated with the principles and operation of the scanning electron microscope. The EB-PBF melting process utilizes a focused electron beam to melt the powder in the bed, which is filled by a hopper and rake combination generally to feed powder to the bed with the electron beam orientation being controlled to shape the desired part, as highlighted in Fig. 11 [[13], [14], [15]].

**Fig. 12:**
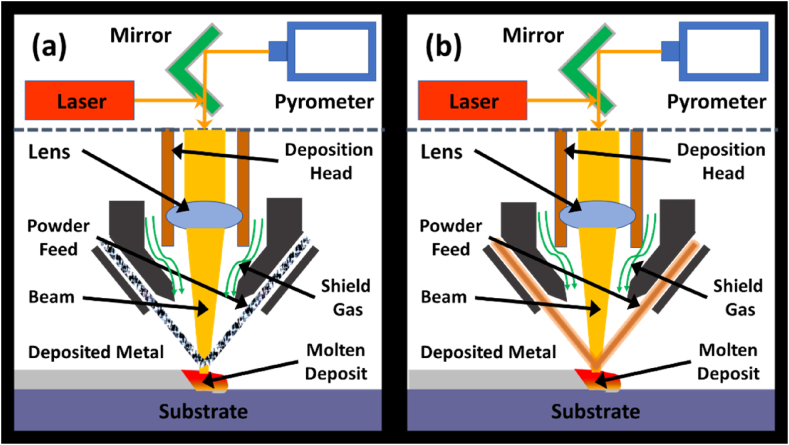
The schematics of Direct Energy Deposition via (a) Powder Material feed, (b) Wire Material Feed.

**The Direct Energy Deposition (DED)** process utilizes an active deposition approach to part forming with the deposition of materials by use of powder feed or metallic/alloy wire in the form of feedstock instead of a powder bed installed in the machine with the heating/melting source in the form of either laser or electron beam [14,16]. The DED process can be categorized into two further categories, as shown in Fig. 12: 1.) The first category deals with the feed in the form of wire (Fig. 12 (a)), which is melted to form layer by layer of the part in the form of welding method, 2.) The second category focuses on using powder flow for part formation and is known as Laser Engineered Net Shaping (LENS) as shown in Fig. 12 (b).

**Fig. 13:**
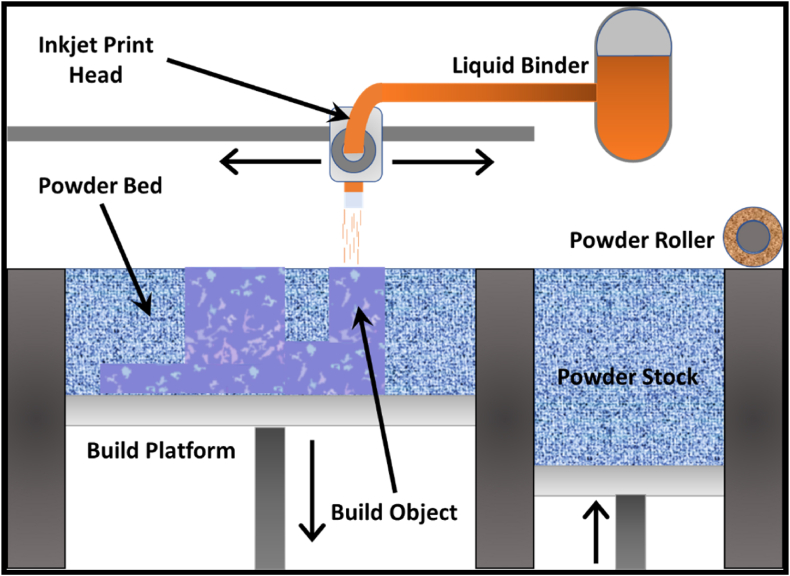
The schematic process of metal binder jetting process.

**Binder Jetting** is the process that utilizes the input of the binder on the top of a powder bed, making it an extension of inkjet printing from 2-D to 3-D. Instead of direct melting of the powder bed, the powder is mixed with a polymeric binder or photopolymer, which is cured under the influence of light and heat to make a near-net shape with a layer-by-layer binding of the polymer to get the desired part. This succeeds by post-processing treatment to remove the binder and gradually sinter it to get the desired dimension, properties associated with the operation and use of the component, highlighted in Fig. 13. It consists of a powder storage tank coupled to an automated sprayer, for even deposition on the powder bed. The action is repeated for each layer using the same deposition, rolling, and scanning principle until the entire part is ultimately achieved. The part obtained is referred to as the green part, which requires additional sintering steps to complete [[17], [18], [19]].

### 1.3 Development of additive manufacturing of IN939

IN939 is one of the more modern alloys developed as a class of nickel-based superalloys that focuses on improved fatigue and creep properties, making it ideal in turbine installations. Their original development method for practical use was based on solid melt casting for gas turbine guide vanes. Further working towards the additive manufacturing process involves investigating the different techniques and parameters associated with them on the final output of the product [[20], [21], [22]].

Several investigations have been undertaken into the influence of process parameters on the microstructure and properties of the final alloy. The primary additive manufacturing process conducted to develop IN939 from either alloy powder or elemental powder has been with the use of LPBF and EB-PBF because of their focused high energy density input sufficient to melt and fuse/weld the raw powder. An exact correlation between the influence of processing parameters and the resultant part properties is not feasible due to slight variations observed in the composition of the IN939 powder procured for the experiment. Furthermore, the instruments utilized along the machine parameter variations lead to variations in the output results associated with microstructure formation and mechanical properties. However, an intricate breakdown regarding the additive manufacturing of the IN939 alloy for the different processes and parametric evaluation is more suitable, which brings us to the point [[23], [24], [25]].

## 2 Production of IN939 parts by additive manufacturing routes

### 2.1 Manufacturing by laser powder bed fusion (LPBF)

LPBF is one of the most explored techniques to produce superalloys, which is critical for providing a high value of irradiance to melt the raw materials. It utilizes a set of optics along with a focusing lens and a high-intensity/energy laser for melting layer by layer of deposited powder on the bed by orientation and movement of the laser as per the part specification [23,26]. IN939 properties are generally well identified for the cast condition, commonly accepted for manufacturing components from the high-temperature alloy [20,22]. However, in recent years, studies on alternate classes of nickel superalloys have shown that additive manufacturing serves as a promising route to improving mechanical properties such as elevated temperature fatigue and creep resistance and optimizing microstructure formation.

The traditional cast alloy route of IN939 involves the two-step solution treatment followed by aging to optimize the alloy's second phase γ′ formation. However, due to the slower cooling rate, the problem is associated with segregation, microstructural inhomogeneities, and undesired phases related to the diffusion of elements. Additive manufacturing eliminates the potential need for solution treatment for homogenizing the alloy due to the short laser interaction time and higher cooling rate of the layer of fused alloy powder. This case is highly coherent with the additive manufacturing of components from nickel-based superalloys, resulting in a few similar microstructural features [27,28]. IN939 has been traditionally the preferred as-cast form. However, developments in additive manufacturing have led to exploration that, coupled with post-processing treatments, can provide a similar level of or superior properties to the cast alloy route.

**Fig. 14:**
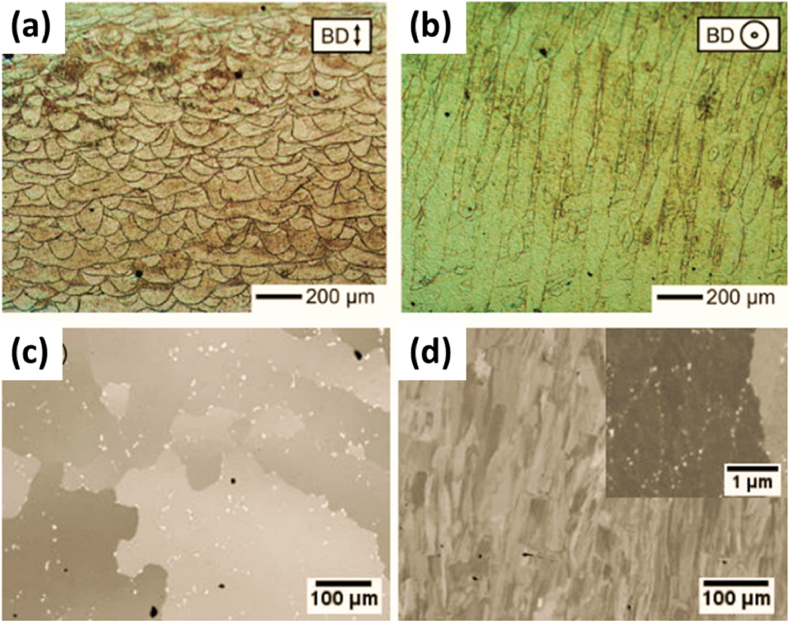
(a, b) Optical micrographs of as-cast sample at 90⁰ specimen with (a) parallel, and (b) perpendicular to the build direction and (c, d) SEM results of the specimens in their as manufactured form, (c) cast, (d) SLM samples [29,41].

**Kanagrajah** explored the idea of LPBF for manufacturing IN939 parts and its properties under static and cyclic loading [29]. On a bed of preheated stainless steel heated to a temperature of 100 °C, the experiment used pre-alloyed powder with a size of 30 μm. Dog bone samples were created using a yttrium fiber laser, with each layer having a thickness of 30 μm. The effect of laser scanning direction on the building sample was also investigated with two orientations being taken as 0⁰ and 90⁰. Multiple LPBF parts were heat-treated along with cast alloys for comparison purposes. The equiaxed structure of grains was observed in the plane of melting, whereas elongated structure was observed in the plane perpendicular to it, as shown in Fig. 14 (a, b) [30,31].

The elongated columnar structure is a recurring feature of the LPBF process regardless of angle. It disappears during the heat treatment process because of recrystallization, which leads to grain growth due to the high amount of distortion energy in the sample because of thermal gradients. The orientation of grains in the LPBF as-build samples was in the [001] direction, showing singular texture dominance, which weakens due to the recrystallization phenomenon during heat treatment [32]. TEM analysis pointed out the coherent distribution of γ′-precipitates in the case of both LPBF and as-cast samples with slightly larger size in the former with the composition determined to be of (Ni …)_3_(Al, Ti, …) type. Additionally, brittle phases were observed in aged LPBF samples, which were found to be η-type precipitates that vary in morphology and composition compared to γ′ degrees [33,34].

Tensile testing of the samples was performed for both 0⁰ and 90⁰ build orientations. It was analyzed in as-build and aged conditions with additional tests run for the 90⁰ exposure after the solution annealing and at a temperature of 750 °C. At RT testing for the as-build infection, the 90⁰ orientation sample provides better performance in terms of flexibility while the yield strength is similar. However, the characteristic performance changes after aging as the 90⁰ samples show higher yield strength with lower ductility [35]. As such, the effect of elongated grains and the preferred orientation of [001] compared to the build direction strongly influence the mechanical properties. The elongation at failure is varied in both orientations by a factor of two, which is attributed to different mean accessible paths for the dislocations to travel due to the difference in grain aspect ratio and orientation of the grain long axis concerning the loading direction [36,37]. It should be clarified here in advance that samples mentioned at 0⁰ are built parallel to the building platform plane, while 90⁰ pieces are made perpendicular to the plane of the built direction.

However, an additional substructure contributes to the samples' overall strength and flexibility, which has been determined to be the low-angle grain boundaries that function as barriers for dislocation motion. Furthermore, both orientations showed elongated and equiaxed substructures, considered sub-micron in size, and influenced the mechanical behavior of LPBF samples in early deformation stages. In the aging treatment, the recrystallization phenomenon annealed out the substructures, which correlated the yield strength with the grain size. As such, with aging treatment, the evolution of concurrent precipitates occurred, which further increased the yield strength and UTS of the samples made in both directions due to the reduced dislocation mobility due to finely dispersed precipitates while the solution annealed samples exhibited properties in between the as-build and the aged conditions due to the initiation of formation of the γ’ precipitates during the process [38,39].

However, unlike conventional alloys, testing at elevated temperatures led to embrittlement due to excess formation of precipitates. This contrasts with the ductility of the as-cast samples, which showed an increase in ductility at high working temperatures because the initial formation of nuclei only occurred during the LPBF process, and, as such, the formation of the precipitates remains inhibited. The fatigue life of all the LPBF samples evaluated at RT was higher than their as-cast counterparts because the stress amplitude generated at 0.55 strain amplitude was 700 MPa, which was higher than the yield strength of the as-cast samples, resulting in premature failure. However, aging led to varying trends whereby fatigue life decreased for the LPBF samples from 4702 h to 1598 h due to increased brittleness because of the precipitates. Still, for the cast samples, it grew from 313 to 2677 h due to the increasing effect of precipitates on yield strength. Porosities could also contribute to decreasing fatigue in life by acting as stress concentrators. However, at elevated temperature fatigue testing, the decrease in fatigue life of LPBF as-build samples is primarily attributed to increased dislocation mobility but increased for the as-cast samples due to acceleration of the formation of precipitates [40].

**Fig. 15:**
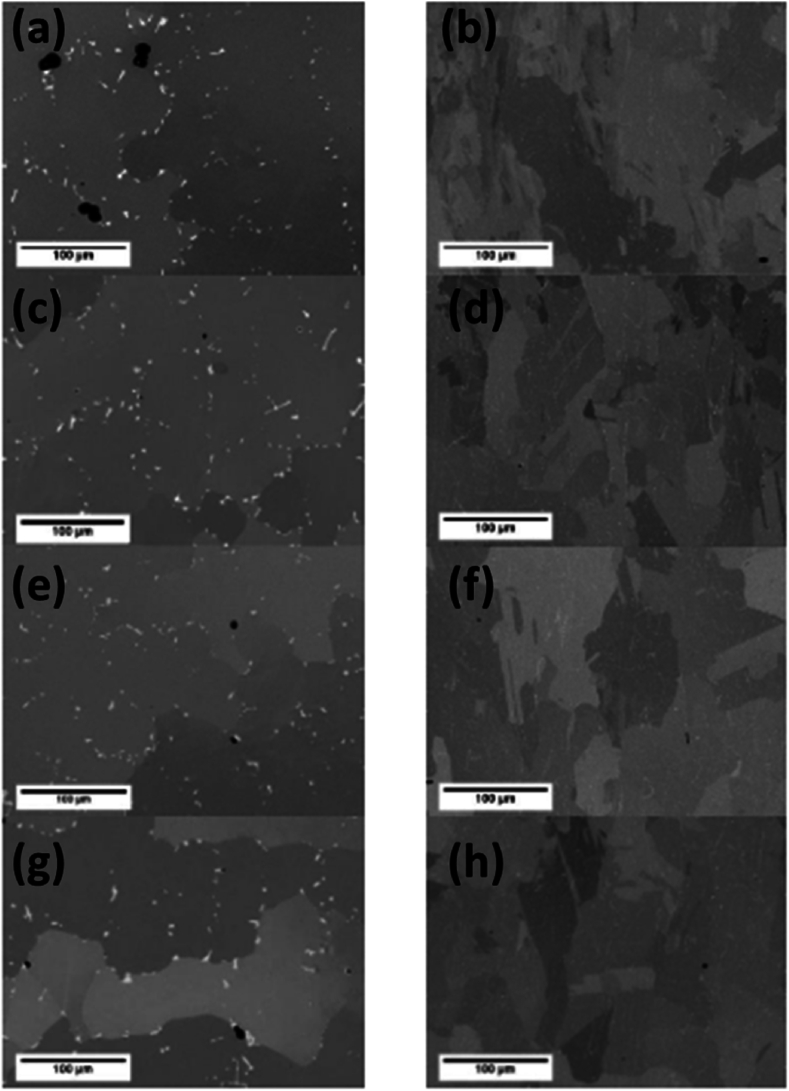
The BSE SEM images of the cast (a, c, e, g) and SLM (b, d, f, h) with (a, b) HIP heat Simulation, (c, d) Solution treatment, (e, f) One Step Aging, and (g, h) Three step aging [41].

**Philpott** also evaluated the comparison of conventional aging treatment between as-cast and selective laser-manufactured components of IN939 alloy using conventional one-step heat treatment and a modified 3-step heat treatment process for achieving the desired with characterization in both the as-build direction of angle 0⁰ and 90⁰ [41]. The as-received and as-build microstructure revealed equiaxed grains, seen in Fig. 14 (c, d) for the former with large precipitates present. In contrast, the LPBF structure revealed a columnar grain structure with a high aspect ratio and fine distribution of precipitates with an average size of less than 50 nm Fig. 15 (b). The microscopy at different heat treatment stages showed that the as-cast material did not develop any significant changes in microstructure. However, thermal stresses contributed to the recrystallization of the sample, leading to an equiaxed structure Fig. 15 (d) [[42], [43], [44], [45]].

The heat-treated samples showed a considerable fraction of carbide formation, which over time decreased as their surface area fraction increased in the cast samples Fig. 15 (a, c). However, for the LPBF samples, the 3-step aging continued expanding the fraction of residues about volume, which can significantly improve creep performance at elevated temperatures, as can be seen from the development of precipitates in Fig. 15 (g, h). The γ’ precipitation did not show much significant difference in both samples except for a slightly larger area percentage of precipitates of LPBF samples after aging. One-step aging produced a significantly larger volume fraction of approximately 20 precipitates per μm^2^ area as seen in Fig. 15 (e, f), which reduced to about seven precipitates per μm^2^ with 3-step aging [46].

Considering these works, **Marchese** investigated the influence of process parameters associated with LPBF on the porosity and the resulting microstructure of IN939 alloy [47]. Cubic samples of 10 × 10 × 10 mm^2^ with fixed laser power of 95 W and 20-μm layer thickness while varying parameters made with varying scanning speed and hatching distance. The volume energy density was varied to determine the densification behavior. A scanning strategy of an angle of 67⁰ was also applied between each layer. The microstructural investigation was performed equivalently to the previous two studies.

**Fig. 16:**
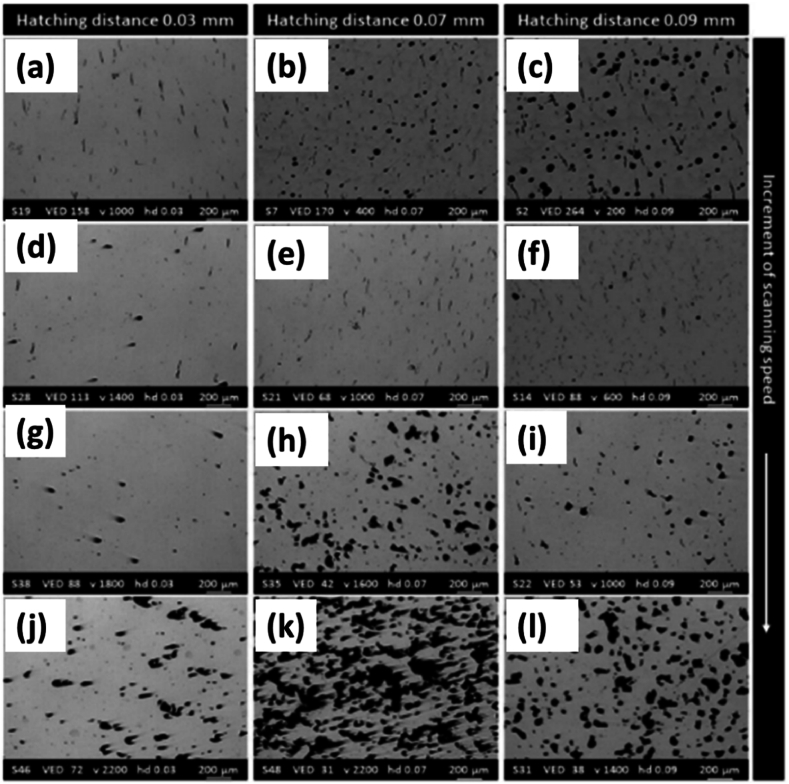
The LOM images of SLM processed IN939samples made using different process parameters such that (a, d, g, j) are hatching distance of 30 μm, (b, e, h, k) represent hatching distance of 70 μm, and (c, f, i, l) represent hatching distance of 90 μm. The scanning speed increasing down the figure such that speed is (a) 1000 mm/s, (b)400 mm/s, (c) 200 mm/s, (d) 1400 mm/s, (e) 1000 mm/s, (f) 600 mm/s, (g) 1800 mm/s, (h) 1600 mm/s, (i) 1000 mm/s, (j) 2200 mm/s, (k) 2200 mm/s, and (l) 1400 mm/s. Note the laser power and layer thickness are constant at 95 W and 20 μm, respectively [47].

The densification results were divided into three ranges. For variable energy density (VED) less than 50 J/mm^3^, the sample showed elevated porosity levels and lack of fusion as insufficient energy was delivered to the powder Fig. 16 (k, l). However, in the range of 50–160 J/mm^3^, the power provided was sufficient to melt, with a small amount of spherical porosity still present and some irregular pores. Still, at greater than 160 J/mm^3^, the melt pool stability occurred, which resulted in keyhole porosity with sizes of pores around 100 μm Fig. 16 (b). The VED also seemingly affected the crack density but without significant trends. Shallow and very high ranges of VEDs, i.e., less than 50 J/mm^3^ and greater than 300 J/mm^3,^ showed a low fraction of cracks because of the more significant presence of other defects as seen in Fig. 16 (e, f, i, j, k, l) [6,48,49].

The hatching distance was inversely related to the densification of the sample, as hatching distances greater than 0.1 mm did not allow significant densification even with slow scanning speeds of 100–400 m/s. For less than 100 μm distance, however, the residual porosity could be minimized to less than 0.5 % in the 800–1000 m/s speed range Fig. 16 (a). Decreasing hatching distance could be coupled with even greater scanning speeds to provide a similar porosity level as seen comparing Fig. 16 (g, h, j, k, l). Still, too low a hatching distance, such as 20 μm, can cause melt pool instability due to overlapping adjacent laser scans [43,50,51].

For all the hatching distances, the scanning speed affected the crack density, with values less than 600 mm/s showing high crack density (Fig. 16 (b, c)), while higher scanning speeds coupled with a small hatching distance mitigate the crack density formation. High rates are associated with low thermal stress incorporation in the sample. No combination of these two sets of parameters could provide a minimum set of porosity and cracking density, but at a hatching distance of 0.03 mm and scanning speeds of 1600–1800 mm/s, the lowest cracking density of <0.4 mm/mm^2^ was found along with moderate porosity of less than 1 %, as seen from Fig. 16 (d, g). As such, the optimal hatching distance coupled with different ranges of scan speeds resulted in low porosity and cracks in samples, some of which could be subjected to further hot isostatic pressing to improve densification further.

The microstructural and EBSD evaluations presented comparable results as seen in previous works, with analysis showing highly elongated grain with a specific texture [001] growth and grain length along 400 μm. Small substructures characterized the dendritic substructures due to overlapping consecutive melt pools. The high-level magnification showed both metal carbides and γ’ precipitates in the microstructure with additional segregation zones due to dendritic regions formation and nuclei formation due to remelting of the powder. The cracking formation and its surrounding morphology suggested strong growth of carbides and precipitates as cracks mainly were formed along the grain boundaries with possibly two mechanisms associated with their formation [22,[52], [53], [54]].

• Liquation cracking
• Strain Age cracking

The cracking mechanisms observed in the IN939 alloy include liquation cracking and strain age cracking, both of which are associated with the growth of carbides and precipitates along the grain boundaries. Recrystallization was found to have a significant impact on the creep properties of the alloy, with the degree of recrystallization affecting the grain size and texture orientation. The presence of carbides and precipitates, particularly γ’, was found to play a significant role in the strengthening of the alloy. Still, prolonged exposure to high temperatures can result in losing strength. The creep properties of the alloy were also affected by the presence of dislocations, elastic modulus, and carbides at the grain boundaries. Mitigation measures for these cracking mechanisms may include controlling the heat treatment processes to minimize the growth of carbides and precipitates, optimizing the grain size and texture orientation through recrystallization, and selecting appropriate temperatures and stresses to avoid the loss of strength and brittle fracture. Overall, a better understanding of these mechanisms and their impact on the material properties is crucial for improving the performance and reliability of IN939 alloy in high-temperature applications.

**Fig. 17:**
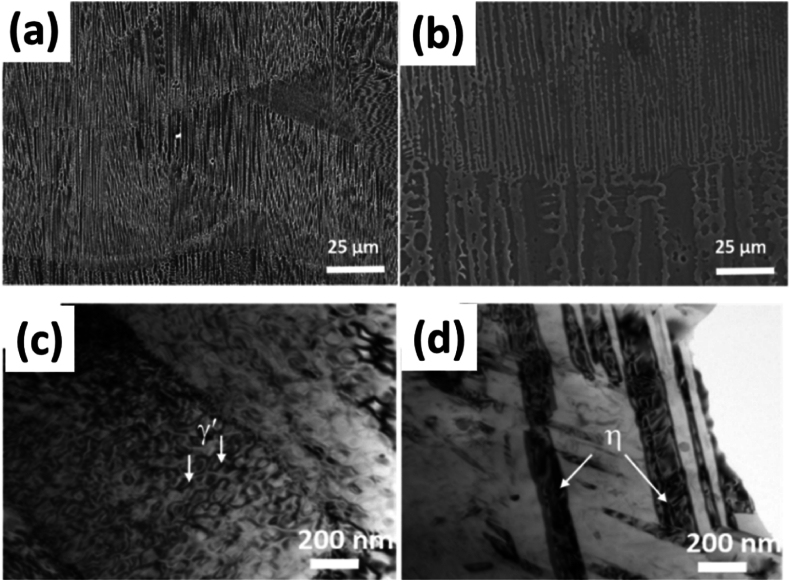
The SEM images of the as-build condition at (a) Higher Magnification, (b) Lower Magnification, STEM images of LTH sample showing (c) gamma prime, and (d) Eta phase [55].

Considering the recrystallization phenomenon occurring inside these alloys, **Banoth** investigated the effect of the recrystallization phenomenon on the creep properties of IN939, which were fabricated as 45 mm cubical specimens and compared to a standard cast sample for reference along with two heat treatments for the LPBF sample [55]. One treatment was labeled as LTH for lower temperature, and the other was taken as HTH for high temperature. However, only LTH treatment was applied to the cast sample. The microstructure of the cast samples exhibited dendritic and inter-dendritic structures, as highlighted in Fig. 17 (a, b). The growth occurred in a specific texture with the [100] direction and featured columnar-shaped grains with higher residual stored stresses. Further analysis revealed many γ′ precipitates and carbides within the matrix of the cast samples Fig. 17 (c, d) [22,56].

**Fig. 18:**
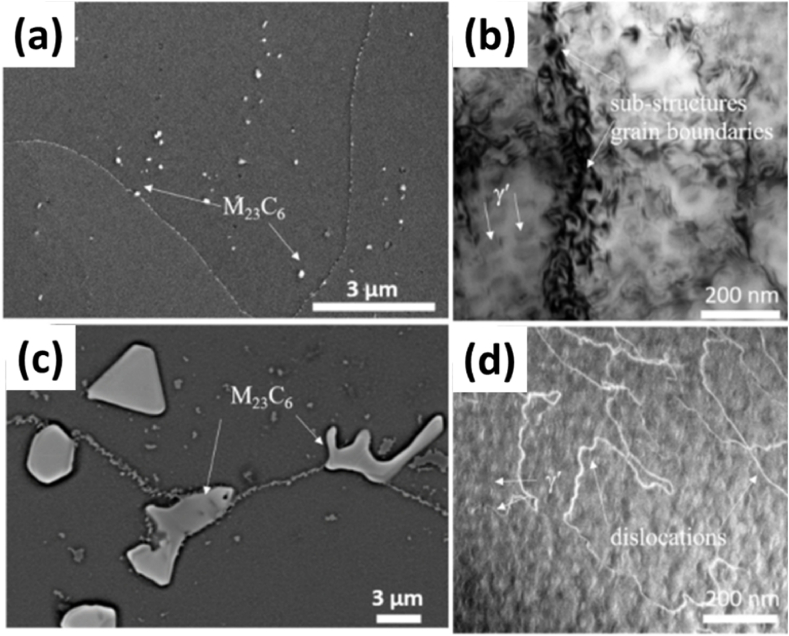
The (a) M_23_C_6_ at the grain boundary. (b) γ. Precipitates, cuboid in shape, and cell-like substructures in the HTH specimen. (c) M_23_C_6_ at the grain boundary. (d) Dislocations and γ? precipitates in the cast LTH specimen [55].

The heat-treated specimens were analyzed for the grain size and the recrystallization that may have occurred. The average grain size of the LTH LPBF and HTH LPBF samples was 27 μm and 50 μm, compared to 32 μm for the cast sample. The recrystallization factor increased compared to the as-build sample, which had 5.75 %, to 12.8 % and 66 % for the LTH and HTH samples, while the cast sample had 99.9 %. After LTH treatment, the as-built texture weakened, and slight recrystallization along with γ′ and η precipitates along with MC and M_23_C_6_ type carbides distributed along the grain boundaries Fig. 18 (c, d). The HTH LPBF samples showed extensive recrystallization, loss of texture, decreased dislocation density, and considerable grain growth to twice the size of the LTH LPBF specimen. The interdendritic regions disappeared entirely due to higher solution treatment temperature, but the presence of MC-type carbides persisted as they are stable up to 1300 °C. The M_23_C_6_ carbides had homogeneous distribution at the grain boundaries and heterogeneously distributed inside the grains along with additional substructures reported by Kangarajah, whose mechanism is not precise in Fig. 18 (a, b). The LTH cast sample had a sizeable equiaxed grain size of 200 μm on average with a recrystallization fraction of almost 100 %, the grain size being four times larger than that of HTH as-build sample along with similar formations of M_23_C_6_ type carbides and γ’ precipitates, as highlighted in Refs. [[57], [58], [59], [60], [61]].

**Fig. 19:**
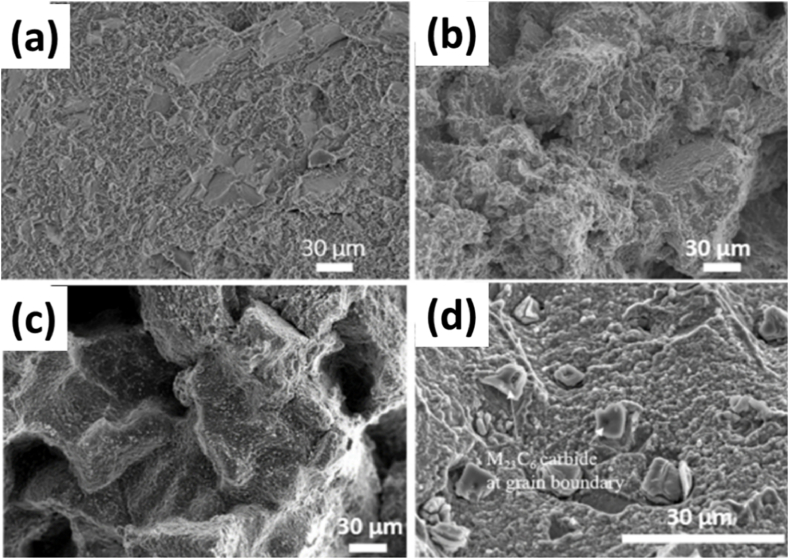
The fracture surfaces of creep-ruptured (a) as-built, (b) LTH, (c) HTH, and (d) cast LTH specimens crept at 816 °C/250 MPa [55].

Creep properties were examined at 816 °C with a stress of 250 MPa. The as-built specimen demonstrated a creep life of 66 h with a strain of 3 % until rupture Fig. 19(a), while the LTH sample exhibited a longer creep life of 203 h, three times that of the as-built sample, with a similar strain. On the other hand, the HTH sample showed more than twice the creep life of the LTH sample at 554 h but with a sharp decrease in creep strain at 0.8 %. However, it was surpassed by the as-cast LTH specimen, which had a creep life of 931 h but reduced elongation at 1.9 %. The impact on the creep properties of these samples is due to two phenomena. The first is recrystallization due to high dislocation density and residual stresses. In the LTH as-built samples, recrystallization of new grains led to grain boundary sliding and crack propagation Fig. 19 (b). The presence of carbides along the grain boundaries in the HTH samples hindered grain growth, forming columnar grains. Consequently, crack initiation, propagation, and fracture primarily occurred along these grain boundaries, resulting in brittle fracture behavior Fig. 19 (c). The presence of these carbides acted as sites for crack nucleation and facilitated their propagation along the boundaries, ultimately leading to the brittle failure of the material. The as-cast LTH sample had a longer creep life due to a comparatively lower dislocation density, but crack propagation and failure were similar to intergranular brittle failure, as highlighted by the fracture surfaces of the specimens in Fig. 19 (d) [60].

The second factor influencing creep behavior is the presence of precipitates, particularly γ′ particles, which are the primary source of strengthening in the sample. However, prolonged exposure to temperatures above 650 °C leads to the transformation of the former incoherent η precipitates, resulting in a loss of strength. The LTH as-built sample exhibited lower creep resistance than the HTH and as-cast LTH samples due to the persistent presence of η precipitates before and after creep testing and the higher concentration of dislocation density. Additionally, the growth orientation of the LTH specimen along the [100] direction resulted in a lower elastic modulus, which negatively affected creep resistance. Conversely, the HTH sample exhibited a mixed orientation, resulting in a higher modulus. Only γ′ precipitates and MC-type carbides were present in the HTH samples, along with recrystallized grains and the absence of the η phase within the matrix. However, the lack of carbides at the grain boundaries in the HTH samples resulted in incoherency between the γ′ particles and the matrix, leading to poor elongation. On the other hand, the LTH as-cast sample demonstrated the most extended creep life due to a combination of factors, including the largest grain size, the lowest dislocation density, and the growth of γ′ precipitates from an average size of 41 nm–156 nm [62,63].

**Fig. 20:**
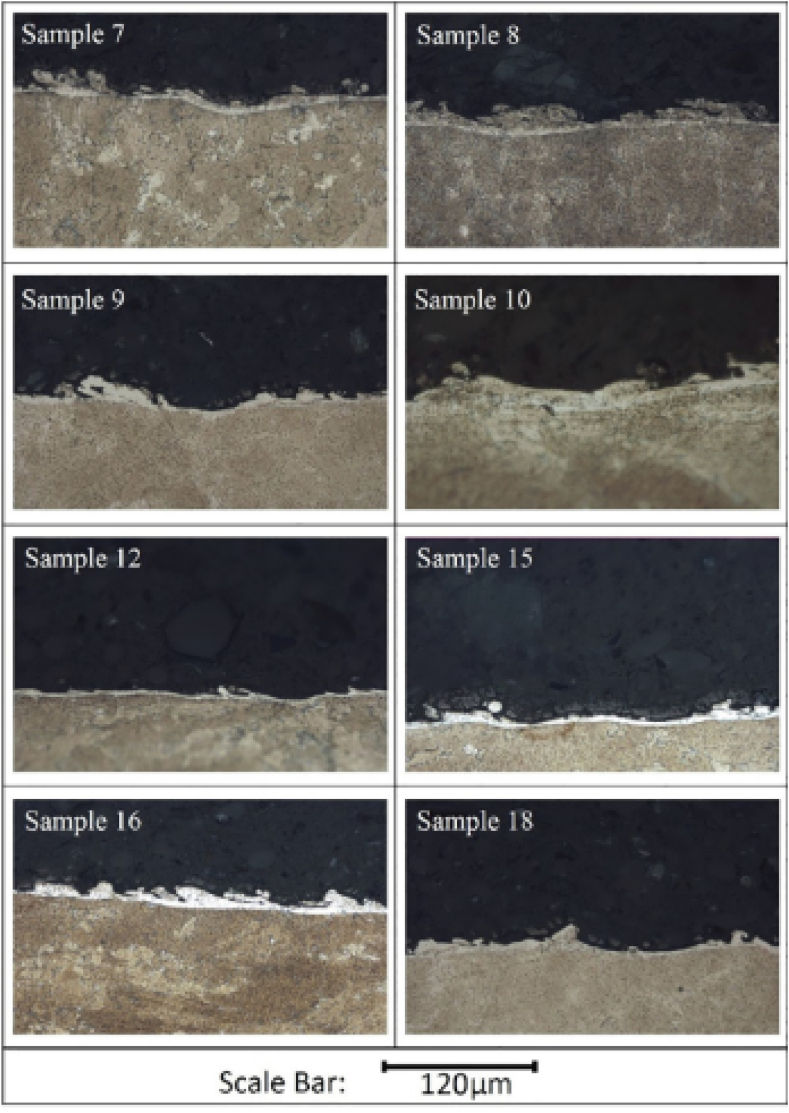
The Optical image of recast layers for some of specimens [64].

In light of the works produced, an extensive gap remains in the parametric investigation IN939 formed by LPBF, particularly the formation of single tracks and the recurring parameters influenced by high-power laser, carried out by **Ozaner** [64]. Multiple samples of 15 × 15 × 15 mm^3^ with the first batch in random scanning followed by the second batch with accurate strategy and variation in laser power and scanning speeds. The morphology of the single track and melt pool formation, as well as the dimensions of the beads, were analyzed, as seen in Fig. 20. Some samples had balling effects and discontinuities in specific locations of the samples due to the lack of sufficient energy being supplied to them. In contrast, other samples had consistent laser tracks without any interferences. Inconsistent balling features were observed for the high scanning speed group (1200–1400 m/s). The balling effect is attributed to the surface tension between the melt pool and the solidified layer, resulting in different solidification behavior, which is further confounded by the instability of the melt pool [65].

The shape of the melt pool was found to be influenced by the energy input from the laser. Lower laser power resulted in shallower and smaller melt pools, while higher power inputs resulted in broader and deeper melt pool geometries. The laser scan speed also played a role, with higher speeds resulting in smaller pool sizes and vice versa. Relative density measurements were performed to assess the influence of scanning speeds and energy on porosity and crack formation in the samples. Increasing the scan speed led to a sharp decline in relative density, indicating insufficient melt formation and the presence of porosity.

The highest density achieved was 99.89 % in sample 12, which utilized a laser power of 300W and a scan speed of 1400 mm/s. On the other hand, excessive pore formation occurred at very high energy inputs due to the evaporation of low melting point elements. This can be observed from the images of the recast layers in Fig. 20 [41,47].

Based on the evaluation of mechanical properties and the influence of microstructure on them, **Shaikh** conducted a study to examine the significance of solution treatment on the as-built microstructure. This study used two types of samples: 15 × 15 × 15 mm³ cubes and cylinders with a diameter of 11 mm and a length of 80 mm, oriented perpendicular to the build direction. Two distinct heat treatments were applied: direct aging and solution treatment, followed by aging. The microstructure analysis confirmed previous findings of elongated columnar grains, dendritic structures, and residues in the interdendritic regions. The particles observed exhibited either a blocky or elongated morphology. The blocky particles are MC-type C, Ti, Ta, and Nb carbides. The elongated particles showed firm peaks of Ni and Ti, along with Cr and Co, indicating the presence of η phase. The samples subjected to aging treatment alone showed bulk precipitation of platelet and spherical particles. The platelet samples were rich in Ni and Ti, with weak additions of Ta and Nb. The spherical precipitates contained Ni, Ti, and Al, confirming them as γ′ precipitates [47,61,66].

**Fig. 21:**
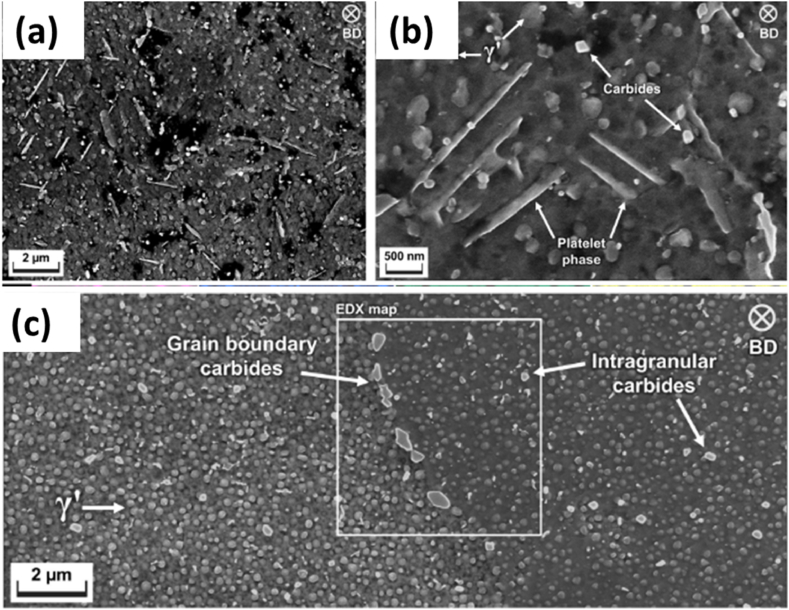
The SEM images of the SLM samples (a, b) Aged without solution treatment, (c) Solution treatment + Aging [83].

In the case of the solution-treated and aged sample, no plate-like phases were observed Fig. 21 (c). The microstructure analysis revealed the presence of well-dispersed intergranular γ′ phases, along with intragranular MC-type carbides. M_23_C_6_-type phases, rich in Cr and W, were also identified at the grain boundaries. On the other hand, the direct-aged sample showed higher yield and tensile strength due to the η phase's presence in the interdendritic region Fig. 21 (a, b). However, it also showed lower strain to failure, likely due to the embrittling effect caused by this phase. These findings were supported by SEMresults shown in Refs. [21,55,67,68].

In light of these developments, a practical setup for the IN939 alloy was developed as a turbine blade fir tree manufactured by LPBF and further followed by solution treatment and then aging by **Ozaner** [69]. The former was conducted to dissolve secondary phases such as γ′ and carbides and the aging to form the fine γ′ phases. The profile of the fir tree was machined from the heat-treated samples. As such, the machining was done based on Taguchi 1.3 experimental design, while the machining parameters were estimated according to Taguchi's signal-to-noise ratio for both EDM and CFG methods used for shaping the part. Three levels of experimentation were conducted for both CFG and wire EDM processes instead of differing parameters as per Taguchi criteria, with further mechanical testing being conducted by microhardness measurement and tensile testing using a unique mounting setup as well as optical microscopy and SEM for microstructural and fracture analysis of the tensile specimens.

**Fig. 22:**
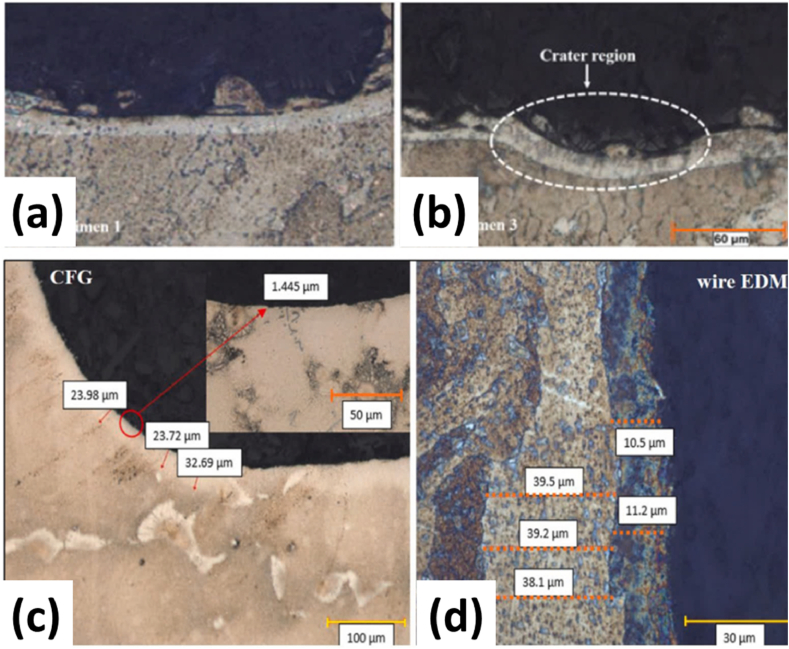
(a, b) The crater thickness and recast region of wire EDM specimens., and (c, d) The optical microscope images of recast layer and HAZ regions for (c) CFG and (d) wire EDM methods [69].

With the varying parameters, the effect on the recast and HAZ layer thickness was observed as well, as highlighted by the optical images in Fig. 22 (a, b). The variation of the wire EDM parameters for shaping the fir tree generated a specific mean pattern for recast thickness and HAZ thickness. In the case of both observations, pulse on and off were the most influencing parameters, followed by the applied voltage. Where pulse on time increased recast and HAZ thickness, pulse off time had the opposite effect. At the same time, the voltage had the same influence as a pulse on time on the recast thickness, whereas, in the case of HAZ, it had a maximum at 120 V of approximately 47 μm. The increase in pulse on or pulse off time led to an increase and decrease in the discharge energy from the operation. This led to an increase in the thickness, microhardness values, and overall profile deterioration.

This is due to the increase in discharge energy, which allowed deeper penetration of increasing temperature into the profile, which increases the HAZ layer thickness formation, and, if not sufficient time is provided for flushing, the molten layer is not filled evenly, resulting in the recast formation. The mean profile accuracy and hardness observed similar non-linear increasing and decreasing behavior with a pulse-on and pulse-off time increase. At the same time, voltage resulted in a minimum for the former and no change in the hardness.

For the CFG method of profile formation, the recast and HAZ thickness values were observed on the samples at both the entry and exit regions of the grinding wheels as shown in Fig. 22 (c, d). The increase in the feed rate led to an increase in both the recast and HAZ thickness, where the depth of cut and wheel speed led to a non-linear, in the case of the former, only for both entry and exit regions. The HAZ thickness increased with both depth of cut and wheel speed for the exit region, while for the entry region, it highlighted local maxima for depth of cut and wheel speed.

**Fig. 23:**
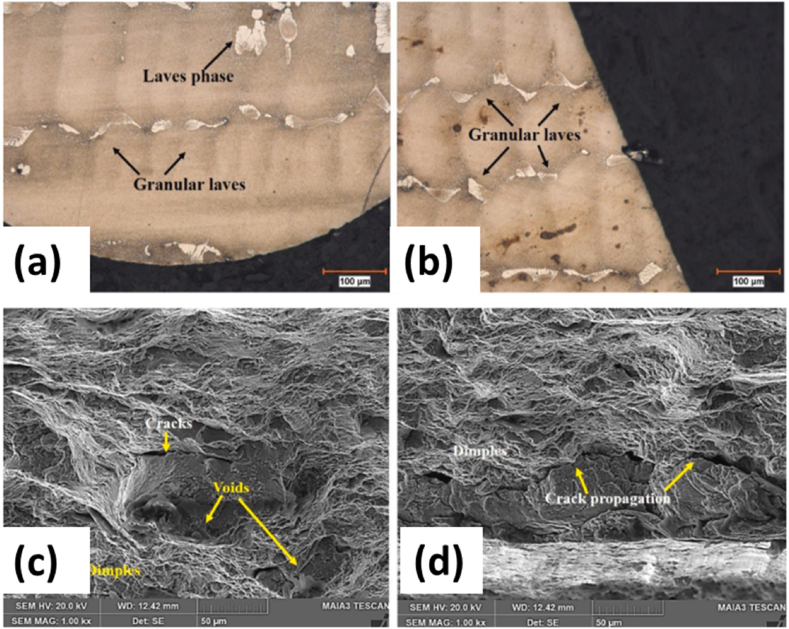
(a, b) The surface images of (Wire EDM) and (b) CFG specimens, and (c, d) The SEM images of the fractures surfaces profiles by Wire EDM [69].

The tensile strength was measured as a function of recast thickness, which found that UTS decreased for both EDM and CFG processes, but higher UTS was observed for the wire EDM-made samples because of higher recast thickness obtained in the samples. However, the recast layer presents crack initiation regions that form via tensile stresses applied on the object, leading to a decrease in the case of CFG specimens. SEM results showed that the region around the edges of the fir tree demonstrated cracks and grain deformation before failure. Still, no deformation was observed between the two fracture surfaces, which showed brittle behavior, as seen in Fig. 23 (c, d). The defective areas observed on the fracture surfaces had trace amounts of Ni elements while being rich in Cr, whereas the matrix was rich in Ni, the defects being dimples, voids, and micro-pores.

The machined specimen surface showed initiation and propagation of intergranular failure cracks and slip bands in all the specimens, which, along with the presence of the laves phase, provide good plastic deformation properties to the specimens Fig. 23 (a, b). EDS showed the presence of chloride impurities at the fracture surfaces, leading to macro-porosity in the sample during sintering because of the volatility of chlorides at feverish temperatures and other problems such as inclusion embrittlement and lower weldability, among others.

### 2.2 Manufacturing of IN939 by other AM processes

Our review has shown that LPBF is the preferred route for additive manufacturing IN939 alloy. However, other methods, such as electron beam-powder bed fusion, direct energy deposition, and binder jetting, are also viable options for fabricating Inconel parts. However, despite extensive technological developments, minimal investigations into these alternate routes have occurred [17,70,71].

**Fig. 24:**
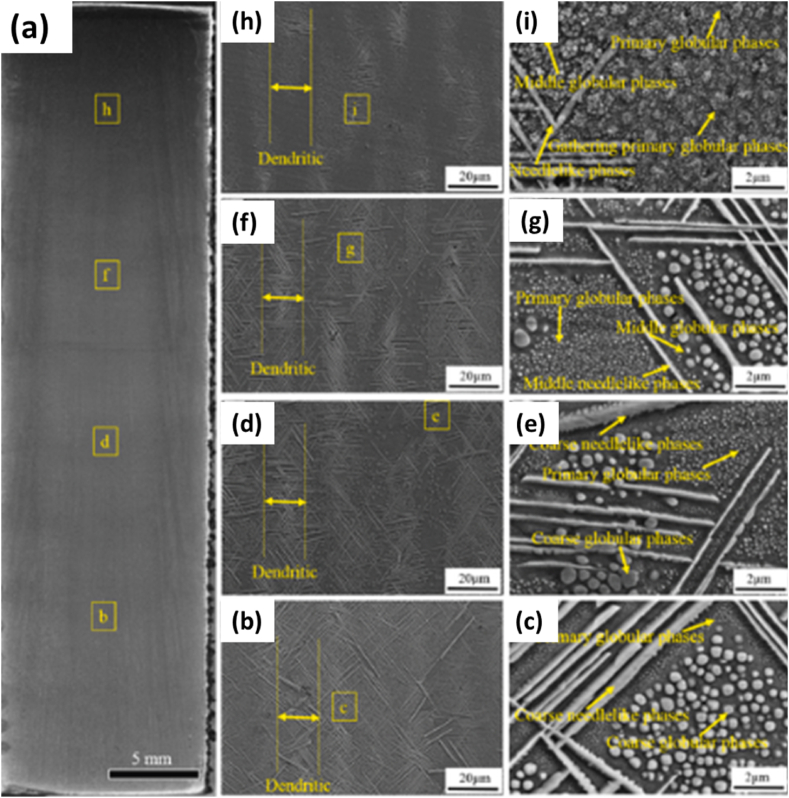
The SEM microstructures of the samples at different heights along the building direction (b, c) 40, (d, e) 30, (f, g) 20, and (h, i) 10 mm [72].

The development of IN939 parts by EB-PBF was conducted by **Li** with a focus on the microstructural and mechanical characteristics of objects [72]. The samples were prepared in a commercial e-beam melting setup. The Z-mode scanning technique was applied with the advancing direction changing by 180⁰ after completing a previous layer with cuboid specimens of 15 mm × 70 mm × 45 mm being prepared for further work. This study applied two post-processing techniques, one involving hot isostatic pressing and the other involving heat treatment. Microstructural analyses were carried out via SEM of the sample at incremental distances from the base in which needle-like and globular phases were observed as shown in Fig. 24 (a). The base's 10 mm and 20 mm microstructure showed coarse needle-like structures and globular phases with additional primary globular in the latter Fig. 24 (b, c, d, e). At 30 mm, middle needle-like structures and globular phases were observed in Fig. 24 (f, g), while at 40 mm, more primary globular phases were observed, and a smaller number of coarse phases were observed Fig. 24 (h, i). As such, the phase size decreased with distance from the base. TEM EDS showed similar compositions for both phases, indicating they were either γ′ or η type. The SAED pattern confirmed the needle-like phase to be η-phase while the globular phase was γ’.

The in-situ heat treatment also occurred in the samples because of the EBSM and preheating of the powder bed. As solidification occurred at 1343.5 °C, the η and γ′ phases precipitated at 1210.9 °C and 1113.9 °C, the main phases per the earlier analysis. This is supported by the fact that IN939 has a higher Ti: Al ratio, which tends to accelerate the precipitation of detrimental η-phase during in-situ or pot processing treatment, either from γ phase of the matrix or the γ′ phase. The heat treatment effect lasted longer at the bottom of the sample during in-situ processing, so it contained a more significant fraction of η-phase. Different durations of in-situ treatment resulted in different microstructures, each transition from primary to middle to coarse phase requiring 3 h. However, as a gradient in the microstructure resulted in a gradient in the microstructure, homogenization becomes crucial. For that, two-step heat treatment involving HIP followed by aging was performed, the first to dissolve the η-phase and the second for the precipitation of finer γ’ precipitates, which were found to be rich in Ni, Al, Ti, and Ta but lacking Cr and Mn [42,73].

The mechanical properties were analyzed by tensile testing of specimens from different heights. The as-built parts demonstrated increasing strength with increasing height owing to the evolving microstructural variations, mainly decreasing volume fraction of η-phase and increasing fraction of finer γ’ phases, as the 40 mm sample experienced better mechanical properties. The yield strength, UTS, and elongation ratio increased after heat treatment due to the dissolution of the η phase and the formation of new finer γ phases. As such, the morphology of the fracture sample transitioned from brittle to ductile failure, with the values of yield strength, tensile strength, and elongation highlighted in Table .3 [42,61,74,75].

**Table 3:** The summarized results of mechanical testing of specimens made via EBSM [72].

S. No	Temperature (⁰C)	Description	Yield Strength (MPa)	Ultimate tensile strength (MPa)	Elongation (%)
1	23	EBSM: 10 mm specimen	627.5 ± 29	900.7 ± 190	7.5 ± 7.3
2	23	EBSM: 20 mm specimen	639.5 ± 25	973 ± 53	11 ± 2.3
3	23	EBSM: 30 mm specimen	653.2 ± 9	960 ± 44	9.6 ± 4.7
4	23	EBSM: 40 mm specimen	667.2 ± 7	967 ± 121	10 ± 5.5
5	23	HIP Specimen	701 ± 4	1153 ± 50	23.9 ± 3.3
6	23	HIP + HT Specimen	810 ± 8	987 ± 116	22.8 ± 8.8
7	25	Cast Reference	703	768	3.3

## 3 Discussion and current research gaps

Developing nickel alloy products has been a critical focus in the additive manufacturing industry. Current work has highlighted recurring deficiencies resulting from the composition of the alloy itself, the process parameters, and the choice of post-processing treatment. Mechanical properties are lost due to a combination of grain anisotropy, detrimental phases, and process-induced defects in the sample. Their presence is influenced primarily by process parameters but also by the raw material itself, particularly the composition of the powder. A systematic overview of developments is necessary given the scope of the limitation of work conducted using IN939.

### 3.1 Comparison of the microstructure

The grain structure has a complex relationship with the additive manufacturing process when considering the process parameters associated with the standard LPBF process and materials properties and the build direction of the sample, all of which contribute to some. IN939 in cast form is generally an equiaxed structure of γ-phase matrix embedded with secondary γ′-phases and possibly η-phase precipitates and MC or M_23_C_6_ type carbides. However, the initial structure seems to vary, as shown in different studies, even if the focus only remains on the LPBF process.

The optical images of the microstructure showed a grain structure distinction when sectioning was conducted parallel or perpendicular to the build direction in the work Kanagarajah. These features are reminiscent of the direction/behavior of melt pool solidification and hence have no broader effect. However, detailed microstructure observation using EBSD showed a slightly lower aspect ratio of grain growth in parallel vs. perpendicular build direction, though both can be considered elongated/columnar grain structures. The result of this variation was more generally attributed to the heat dissipation route/mechanism in both directions during the solidification stage. This resulted in a combined average grain size of 70 μm for the 0⁰ build direction and 35 μm for the 90⁰ build direction. Relative to the as-cast sample, however, the grain size for the build samples was smaller by a factor of 3.

Philpott also reported a similar columnar grain structure in the as-build LPBF sample. He further confirmed the extensive recrystallization in the as-build sample during solution and annealing treatments. This led to an equiaxed structure and the growth of new second-phase MC-type carbides rich in titanium and carbon. This was not clarified, given the heat treatment details were not presented in the work. However, it was clear that these carbides were formed early in the treatment at the pre-existing grain boundaries before recrystallization and remained in position. In this study, they exercised two different heat treatments, which saw that the 3-step aging treatment led to a higher increase in count per area. Similarly, prolonged 3-step aging treatment leads to complex growth of γ’ precipitates while decreasing their quantity [41].

The observation of Marchese work on optimized samples using the VED approach showed textured as build LPBF samples along the (001) orientation when a sample with the lowest cracking density was analyzed, this being like the observation of Kanagrajah with a typical cross-section size of 35 ± 15 μm along the x-y plane and length reaching 400 μm. Also observed were primary dendritic regions along with segregation and precipitation along the cracks in the inter-dendritic region. EDS showed these were likely MC carbides or γ’ precipitates given Nb, Ti, and C enrichment compared to other elements [30,55].

Further affirmation of the present microstructure of the as-built LPBF samples was provided in the work of Banoth, who stated the development of dendritic and inter-dendritic regions as well as columnar grain formation in the (100) due to epitaxial layer growth with the average grain size determined to be 32 μm. A key influencing factor in the microstructure design has been the treatment temperature by Banoth. The work uses different treatments on LPBF samples with variations in solution treatment temperature. The higher solution treatment temperature of 1240 °C in the HTH treatment led to an increased recrystallization factor of 66 % while the average grain size increased to 50 μm [37,76,77].

Despite the higher recovery and recrystallization at this temperature, elongated grains persisted. This is due to the pinning effect of carbides along the grain boundaries, which were 100 nm in size. The dissolution of carbides occurs above 1300 °C effectively, so further heat treatment at that temperature range is to be considered for complete grain growth in the lateral direction. These carbides were M_23_C_6_ types, which are comparatively more minor and segregate at the grain boundaries uniformly and heterogeneously inside the grain. These coincide with the latest work conducted by Shaikh concerning the development of the heat treatment cycle to determine the necessity of the solution treatment step. The as-built microstructure contained a columnar structure with dendritic regions and blocky and elongated particles. The former is confirmed to be MC-type carbides, while the latter are found to be η-phase particles. Direct aging of the as-build has provided a microstructure comprising elongated and spherical γ′ particles. However, with the use of solution treatment, the platelet particles disappeared. In addition, the γ’ particles were dispersed in the matrix along with the presence of intergranular MC carbides and grain boundary M_23_C_6_-type carbides.

**Fig. 25:**
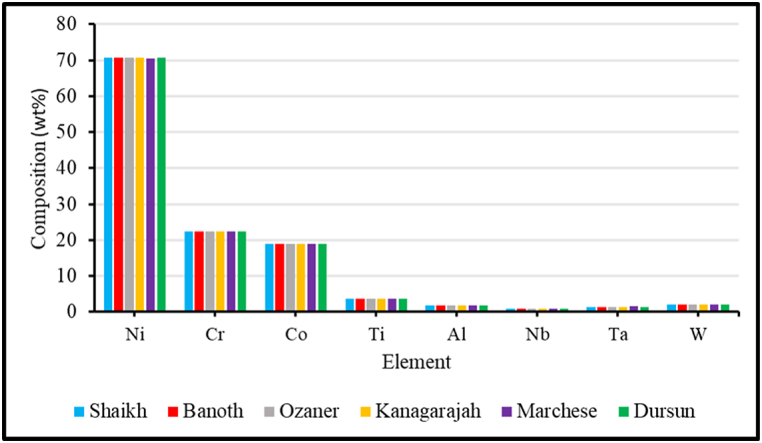
Comparison of powder elemental composition used in different additive manufacturing studies of Inconel 939.

Another critical aspect that has been debated is comparing the composition of IN939 compositions utilized in the alloy. As can be seen from the graph of the comparison of IN939 compositions mentioned in each study, the percentage of elements is, if not wholly, similar. The elements C, B, and Zr have been removed because they were similar in all studies or because their relative composition is too minuscule to be considered for discussion, as seen in Fig. 25. The similarity in composition negates the influence of composition difference hence, the determining parameters are associated with the equipment and consequently with the heat treatment cycles applied.

The limited amount of work highlights some certainty regarding the treatment of this alloy. The use of solution treatment is critical, as highlighted by Shaikh, as it helps to eliminate the deleterious η-phase in the matrix and substructures during the cooling phase [61]. The growth of γ′-phases is impossible in the case of LPBF samples during manufacturing since the cooling rates are incredibly high (106 °C/min), inhibiting diffusion and segregation of elements at the grain boundaries [[78], [79], [80]]. However, the presence of carbides and an elongated structure can be problematic. Although no work has been done to address this issue, it may be possible.

It is possible to achieve near-cast densification of the IN939 alloy by employing specific techniques. One approach involves utilizing Hot Isostatic Pressing (HIP) treatment to address residual cracks, porosities, and carbides. The HIP treatment helps eliminate these defects and improve the overall density of the alloy. A practical solution treatment near the carbide dissolution temperature is recommended after the HIP treatment. In cases where a HIP facility is unavailable, performing a homogenization step as an alternative may be advisable. However, it should be noted that the effectiveness of this step might not be as high as HIP treatment in eliminating defects and achieving densification. Therefore, if possible, HIP treatment is the preferred option. An aging treatment is recommended to enhance the alloy's mechanical properties further. This treatment allows for the controlled growth of the γ′ phase, the primary strengthening component. The specific holding temperature and time during the aging treatment depend on the γ particles' desired size. Maintaining the size of the γ′ particles in the nanoscale range is crucial to ensure practical dislocations and matrix pinning during the deformation process. Overall, by employing HIP treatment to eliminate defects and carbides, followed by appropriate solution treatment and aging, near-cast densification of the IN939 alloy can be achieved while optimizing its mechanical properties.

### 3.2 Comparison of mechanical properties

The issue with the evaluation of the mechanical performance of IN939 via additive manufacturing is there have been very few works carried out on this alloy, and even less has been done in this regard with a narrow approach utilized.

Generally, in trying to achieve desirable properties in additively manufactured components, the ideal aim regarding achieving mechanical properties is the same as those achieved in as-cast alloys. The properties are available in the literature and provide reliable information concerning the development of properties, including yield strength, tensile strength, hardness, fatigue, and creep, as well as an outline of variation of tensile properties with temperature.

The work of Kangarajah explores the tensile properties of these alloys. However, the yield strength could not be calculated due to poor resolution and no mention. In contrast, the ultimate tensile strength was considerably lower than the UTS provided by suppliers, which is approx. 1500 MPa, but the maximum tensile strength achieved was for the RT 90⁰ annealed sample of 1293 MPa with an elongation close to 12.7 %, similar to that found in cast alloys. However, given the UTS achieved, it is conceivable that the yield strength would be lower, which would be further depleted when servicing the part at a higher temperature. Although the elongation to failure for the as-build sample in the 90⁰ built was significantly higher than usually found at 24.9 %, the UTS was measured for the treatment at 1032 MPa. This work showed that the post-processing treatments were insufficient to achieve as-cast mechanical properties [29,54,81,82].

The only other works that have evaluated the tensile properties are those carried out by Ozaner and Shaikh. However, their work has been limited to specific aspects. Ozaner's work focuses on the influence of Wire EDM parameters on the tensile properties. Although it can be argued that the parameters utilized for processing the alloys provide a higher tensile strength (1340–1470 MPa), it does not provide a complete overview of its mechanical behavior. On the other hand, the tensile results provided by Shaikh showed that proper heat treatments can help achieve near or perfect cast mechanical properties. However, they lacked strain to failure, achieving only a maximum of 6.4 %, half of the failure strain in the as-cast condition. The current issue with fatigue testing is that only low cycle fatigue evaluation has been carried out by Kanagarajah at a temperature of 750 °C, with a strain amplitude of 0.5 %, a strain rate of 6 × 10^−3,^ and an R ratio of −1 [64,83].

The work showed that the fatigue life for the as-built sample was highest at 4702 cycles, which is unacceptable for long service life. On the other hand, creep analysis has reported creep Banoth, who demonstrated the effect of recrystallization on the creep life of the samples.

The high-temperature heat treatment (HTH) showed a superior creep life of 554 h, 2.7 times that of the low-temperature heat treatment (LTH) sample, albeit with a drastic decrease in strain to failure of only 0.8 %. The LTH samples exhibited a superior creep life of 203 h compared to the as-built sample's 66 h, with a high strain of 2.7 %. The primary contribution to the high strain rate can be attributed to factors inhibiting crack propagation along the grain boundaries, which were determined to be carbides on the boundaries in the size range of 1–10 μm, in addition to intergranular ductile failure. Conversely, the HTH samples exhibited intergranular brittle failure. The investigation does not provide details on the low strain rate of failure for the HTH sample, although the low grain boundary area can be considered the reason since the average grain size of the LPBF HTH sample was twice that of the LTH sample. Therefore, a more detailed and systematic approach is required to study the creep properties of these alloys, determining the appropriate parameters and test temperatures after establishing the ideal heat treatments [55,[84], [85], [86]].

The HTH treatment showed a superior creep life of 554 h, 2.7 times that of the LTH sample, albeit with a drastic decrease in strain to failure of only 0.8 %. The LTH samples showed a superior creep life of 203 h compared to the as-built sample, which is 66 h and at a high strain of 2.7 %. The primary contribution to the high strain rate can be taken from factors inhibiting crack propagation along the grain boundaries, which were determined to be carbides on the boundaries in the size range of 1–10 μm, and the intergranular ductile failure. The HTH samples, on the other hand, exhibited intergranular brittle failure. The investigation does not detail the low strain rate of failure for the HTH sample, though the low grain boundary area can be considered the reason since the average grain size of the LPBF HTH sample was twice that of the LTH one. Therefore, a more detailed-oriented approach is required to work on these alloys' creep properties more systematically to determine the appropriate parameters and test temperatures after determining the ideal heat treatments [[87], [88], [89]].

This highlights the necessity of maintaining the scan speed, laser power, and hatching distance, as well as the temperature and service time of samples during post-processing heat treatments, under the following constraints.

• Heating Rate
• Cooling Rate
• Cycle steps
• Temperature
• Holding Time
• Atmosphere
• Tensile, creep testing parameters

The heating rate is not clearly defined in most recent studies for heat treatments. However, considering the limitations associated with equipment operations and safety aspects, the literature and equipment capabilities will set the heating rate. The minimum heating rate found in the literature is three °C/min, which can be considered a benchmark for assessing the heating rate of the samples. The cooling rate depends more on the cooling environment required for the heat treatment process. In particular, the treatments associated with the aging treatment of the samples involve natural cooling, furnace cooling, or convection cooling, which are most likely employed to develop the required microstructure and precipitation. During the heating, holding, and cooling processes, an inert atmosphere is necessary, so the chamber is fully purged with argon to prevent oxidation of the sample and the formation of brittle inclusions and phases [18,90]. The mechanical evaluation has primarily focused on tensile and creep testing for the specimens, along with Vickers hardness testing.

The gauge length and sample dimensions highlighted in different studies did not specify the standard utilized in sample preparation or testing parameters. Shaikh provided a standard for conducting tensile tests of specimens at room temperature using ISO 6892-1 in crosshead mode with a strain rate of 0.00025/s and 0.002/s until fracture [83]. At the same time, the dimensions of the machines were designed according to benchmarks set in ASTM E8M standard for round machined tension test specimens with a gauge length of 25.4 mm and gauge diameter of 4.75 mm. Three samples were tested for each heat treatment condition. In addition, a comprehensive survey of the efforts in highlighting the mechanical properties of additively manufactured IN939 is lacking, with the full scope of evaluation highlighted in Table 4, except the work done by Ozaner due to exceptional variation caused by parameters.

**Table 4:** Summary of mechanical tests performed for different studies done for IN939 via LPBF.

Machine Type	Condition	Orientation	Yield Strength (MPa)	Ultimate Tensile Strength (MPa)	Elongation (%)	Hardness (V)	Fatigue Life (Cycles)	Creep Life (h)	Reference
LPBF 250	As-Built	RT 0⁰ as built	NA	971.5	14.1	NA	4702	NA	29
		RT 90⁰ as built	NA	1032.4	24.9	NA		NA	
		RT 0⁰ aged	NA	1003	0.855	NA	1598	NA	
		RT 90⁰ aged	NA	1237.9	1.26	NA		NA	
		RT 90⁰ ann.	NA	1293.1	12.7	NA	NA	NA	
	Heat Treated	750 °C as built	NA	747.1	1.26	NA	209	NA	
		750 °C aged	NA	1045.1	1.14	NA	73	NA	
		750 °C ann.	NA	801.1	6.1	NA	NA	NA	
EOS M270	As Built	XY	NA	NA	NA	NA	NA	NA	41
		XZ	NA	NA	NA	NA	NA	NA	
	HT 1	XY	NA	NA	NA	NA	NA	NA	
		XZ	NA	NA	NA	NA	NA	NA	
	HT 3	XY	NA	NA	NA	NA	NA	NA	
		XZ	NA	NA	NA	NA	NA	NA	
CONCEPT MLAB Cusing R System	As Built	XY	NA	NA	NA	NA	NA	NA	47
		ZY	NA	NA	NA	NA	NA	NA	
EOS M290	As Built	ZY	NA	NA	NA	NA	NA	66	55
			NA	NA	NA	NA	NA		
	Heat Treated	ZY LTH	NA	NA	NA	NA	NA	203	
		ZY HTH	NA	NA	NA	NA	NA	554	
EOS M290	As Built	XY	NA	1431	NA	NA	NA	NA	64
		Z	NA	1414	NA	NA	NA	NA	
EOS M290	As Built	NA	NA	NA	NA	NA	NA	NA	83
		NA	NA	NA	NA	NA	NA	NA	
	HT 1	NA	1214	1429	4.6	NA	NA	NA	
							
	HT 2	NA	1149	1438	6.4	NA	NA	NA	

## 4 Conclusion

Inconel 939 has been only more recently explored for advanced application via additive manufacturing. This has led to an increase in the further exploration of components and the influence of process parameters on this age-hardenable alloy. However, the as-built IN939 samples often exhibit residual thermal strains, stresses, and porosity, which can negatively affect their properties. Post-processing treatments have been identified to address these challenges as essential for homogenizing the samples, controlling their microstructure, and minimizing porosity. Despite the benefits of these treatments, they are often multi-step and require complex cycles to develop the desired phases. These advancements in AM and post-processing techniques are expected to widen the range of applications for IN939 parts and increase their performance in extreme environments. However, in addition to optimizing the microstructure and mechanical properties of IN939 parts. By understanding the behavior of IN939 parts in extreme environments instead of the present developments, researchers can develop more targeted approaches to optimize their properties and expand their potential applications. Integrating Hot Isostatic pressing at different stages of the multi-step heat treatment process with optimized parameters can aim to provide a component with a homogeneous microstructure and superior isotropic mechanical properties.

## Funding

This work has received funding from the European Union's 10.13039/501100007601Horizon 2020 research and innovation program under the Marie Skłodowska-Curie grant agreement No 101034425 for the project A2M2TECH and The Scientific and Technological Research Council of Türkiye (TUBITAK) supported by the projects coded "120C158″ within the scope of TUBITAK's 2236/B program.

## Data availability statement

The authors would like to indicate that no data was used in the manuscript.

## CRediT authorship contribution statement

**Syed Abbas Raza:** Writing – review & editing, Writing – original draft, Visualization, Methodology, Conceptualization. **Olcay Ersel Canyurt:** Writing – review & editing, Validation, Supervision, Project administration, Methodology, Conceptualization. **Huseyin Kursad Sezer:** Writing – review & editing, Validation, Supervision, Project administration, Methodology, Conceptualization.

## Declaration of competing interest

The authors declare the following financial interests/personal relationships which may be considered as potential competing interests:

Prof. Olcay Ersel Canyurt reports financial support was provided by 10.13039/501100000780European Commission. Prof. Olcay Ersel Canyurt reports financial support was provided by 10.13039/501100004410Scientific and Technological Research Council of Turkey. If there are other authors, they declare that they have no known competing financial interests or personal relationships that could have appeared to influence the work reported in this paper.
